# Exploring the link between fat-soluble vitamins and aging-associated immune system status: a literature review

**DOI:** 10.1186/s12979-025-00501-3

**Published:** 2025-02-17

**Authors:** Hendrik Schmieder, Christian Leischner, Alban Piotrowsky, Luigi Marongiu, Sascha Venturelli, Markus Burkard

**Affiliations:** 1 Department of Nutritional Biochemistry, University of Hohenheim, Garbenstraße 30, Stuttgart, 70599 Germany; 2 Department of Vegetative and Clinical Physiology, Institute of Physiology, University of Tuebingen, Wilhelmstraße 56, Tuebingen, 72074 Germany

**Keywords:** Fat soluble vitamins, Vitamin A, Vitamin D, Vitamin E, Vitamin K, Immune system, Elderly, Recommended Dietary Allowance, Immunomodulation

## Abstract

The importance of vitamin D for a well-functioning immune system is becoming increasingly evident. Nevertheless, the other fat-soluble vitamins A, E and K also seem to play a central role regarding the adequate function of immune cells and to counteract excessive immune reactions and inflammatory processes. However, recognizing hidden hunger, particularly micronutrient deficiencies in vulnerable groups like the elderly, is crucial because older adults often lack sufficient micronutrients for various reasons. This review summarizes the latest findings on the immune modulating functions of fat-soluble vitamins in a physiological and pathophysiological context, provides a graphical comparison of the Recommended Daily Allowances between Deutschland, Austria, Confoederatio Helvetica (D-A-CH; eng. GSA, Germany, Switzerland, Austria), Deutsche Gesellschaft für Ernährung (DGE; eng. German Nutrition Society) and National Institutes of Health (NIH) across all age groups and, in particular, addresses the question regarding the benefits of supplementation of the respective micronutrients for the aging population of industrialized nations to strengthen the immune system. The following review highlights the importance of fat-soluble vitamins A, D, E and K which play critical roles in maintaining immune system function and, in some cases, in preventing excessive immune activation. Therefore, a better understanding of the relevance of adequate blood levels and consequently potential supplementation strategies may contribute to the prevention and management of infectious diseases as well as better overall health of the elderly.

**Supplementary Information:**

The online version contains supplementary material available at 10.1186/s12979-025-00501-3.

## Background

The world population is gradually increasing and reached a record high of more than eight billion people in November 2022 [1]. According to the United Nations, people aged 65 and older, comprise the fastest growing age group and it has been estimated that the global number of older residents will be doubled by 2050 (2.1 billion people over 60 compared to 1 billion in 2020) with additional five years of life expectancy at birth due to improved medical care and public health interventions [1–3].

Aging is a rather individual yet inevitable, time-dependent process of physical degradation that is being characterized by increased cellular and molecular damage, along with alterations of the body composition, organ functionality and resilience against multiple stressors [2, 4]. Other important aspects include for example genome instability, telomere attrition, epigenetic alterations, cellular senescence, chronic inflammation and dysbiosis that can be collectively referred to as “hallmarks of aging” [5]. Other than that, aging is associated with a physical and functional decline of components of the innate as well as adaptive immune system, as part of a distinctive immunomodulatory process called immunosenescence due to antigen exposure and oxidative stress [6, 7]. It is accompanied by an altered composition and functionality of immune cell subsets including circulating monocytes, dendritic cells (DCs), neutrophils, B cells and T cells along with a state of chronic inflammation (inflammaging) [7]. Another aspect of aging relates to the restricted cell and tissue renewal along with dermal and subcutaneous atrophies in older adults, which leads to reduced protection against pathogen invasion as a result of compromised physical barriers such as the skin, mucus membranes or gut epithelium [8]. It has been shown that people aged 60 and older exhibit lower levels of secretory immunoglobulin (Ig) A which is essential for the defense against mucosal pathogens [9].

Moreover, innate immune receptors such as toll-like receptors (TLRs) or nucleotide-binding oligomerization domain (NOD)-like receptors (NLRs) detect signs of cell death or damage, for example nucleic acids or mitochondrial DNA, which causes the release of pro-inflammatory cytokines [10]. Contrary to that, older people often exhibit a reduced activation of the adaptive immune response triggered by the innate immune system [11]. In line with that, hematopoietic stem cells mature and hematopoietic tissue decreases, resulting in a mitigated lymphocyte production [12, 13].

The composition of immune cell populations as well as their effector functions like pathogen clearance or phagocytosis changes during the process of aging, which is delineated for instance by an increased number of memory T cells compared to naïve T cells due to thymus shrinkage, hence the loss of lymphoid tissue [11, 14, 15]. As a result, the immune function of the elderly becomes impaired, which concurrently increases their susceptibility towards certain medical conditions like infectious diseases, cancer or autoimmune disorders [8, 16].

One potential explanation encompasses the already mentioned continuous exposure to antigen stimulation as well as oxidative stress entailing the production of reactive oxygen species (ROS), which is of great relevance for immune cells with a large amount of polyunsaturated fatty acids (PUFAs) in their plasma membrane, since this makes them vulnerable to lipid peroxidation, causing a loss in plasma membrane integrity which ultimately results in a compromised immune response [13, 17–21]. Equivalent to that, age-related changes occur in certain signaling pathways, such as the mammalian target of rapamycin (mTOR) pathway, concomitantly leading to immune cell dysfunction [22].

Another potential molecular mechanism involved in immune aging comprises telomere shortening, which occurs during immune cell division and differentiation. Such a shortening might cause DNA damage or cell cycle arrest, leading to reduced pathogen clearance due to the functional loss of the aforementioned immune cells [16, 23, 24]. Moreover, senescent immune cells of various organs release pro-inflammatory cytokines (for example tumor necrosis factor α (TNF-α), interleukin 1 (IL-1) and IL-6), chemokines or matrix metalloproteinases, which contribute to the development of a rather pro-inflammatory phenotype, the so-called senescence-associated secretory phenotype (SASP), causing DNA damage and a state of chronic systemic lowgrade inflammation [8, 16, 25–29]. Potential consequences for the elderly include, for example, tissue damage, impaired immune responses (immunosuppressive microenvironment) and disrupted tissue environments, leading to the development of numerous age-related disorders [26, 29–31].

In accordance with this, aging-associated immunologic alterations comprise reduced pathogenic resilience, impaired development of long-term immune memory and declined vaccine efficacy, alongside an increased susceptibility to viral infections with exacerbated symptoms and an overall deteriorated etiopathology, as this heightened vulnerability of older people, especially of those with comorbidities, has been highlighted by the recent coronavirus disease 2019 (COVID-19) pandemic [7, 32–34]. A decline in immunogenicity induced by vaccination has been shown by Müller et al. [35]. More than 30% of people aged 60 and over showed no detectable neutralizing antibodies after the second dose of the BNT162b2 COVID-19 vaccine, developed by *BioNTech* (Germany) *and Pfizer* (USA), in comparison to the younger group where almost 98% exhibited a significant immune response defined by antibody production [35]. A similar observation has also been made for the seasonal influenza vaccine, where the elderly exhibited a significant reduction of immunization protection (17–53% protection) compared to younger adults (70–90% protection) [36]. Influenza-related morbidity and mortality might be caused by the aforementioned physical and immunological alterations, exposing older people to an increased risk of secondary bacterial infections of the respiratory tract (bronchitis, bacterial pneumonia), which is the reason why, according to the World Health Organization (WHO), the majority of influenza-associated deaths in industrialized nations occur among the elderly aged 65 and older [37, 38].

In addition to the aging process, overweight and obesity can negatively affect immune function and elevate the risk of infections. This is primarily attributed to a higher prevalence of micronutrient deficiencies in these populations, highlighting the need for an adjusted nutrient intake [39–41].

Another important issue associated with decreased immune function in older adults is micronutrient deficiencies, as well as the less pronounced form, namely micronutrient inadequacies, which represent distinct forms of malnutrition [42, 43]. Malnutrition per se contributes, for example, to an increased intensive care unit (ICU) mortality rate among hospitalized patients [44, 45]. It is believed, that there is a bidirectional relation between nutrition, hence an adequate micronutrient status, infection and immunity, as an inadequate supply with vitamins and minerals increases the susceptibility to infectious diseases which in turn causes the proceeding malnourishment with said micronutrients due to an elevated demand and restricted intestinal absorption [32, 46]. In conjunction with that, vitamin A (retinol), vitamin B_2_ (riboflavin), vitamin B_6_ (pyridoxin), vitamin B_9_ (folate), vitamin B_12_ (cobalamin), vitamin C (ascorbate), vitamin D (calciferol) and vitamin E (tocopherol), just to name a few, downrightly contribute to a properly functioning immune system [33, 47]. Notwithstanding, extensive scientific research studies on the micronutrient serum status in older adults are scarce due to high expenditure, partly because of methodological problems such as deficiency screening or the absence of appropriate markers of stored or available micronutrients [42, 48].

Nevertheless, approximately 35% of the people in India, Europe, USA, and Canada aged 50 and older exhibit an insufficient status of at least one of the essential micronutrients [49]. Vitamin, mineral and trace element deficiencies, also referred to as “hidden hunger” have been described as a global problem which concerns more than two billion people, especially vulnerable population groups like the elderly [50, 51]. One reason for that might be the inadequate food intake, a phenomenon called anorexia of aging that among other factors is caused by physiologic changes such as decreased olfactory function, odontogenic conditions or dysphagia, leading to an overall reduction of food intake alongside poor food choices with an ubiquitous lack of nutritious value and variety, due to anhedonia of eating [51–53].

Moreover, older people tend to exhibit a decrease in intestinal absorption and utilization of vitamins, an example being the reduced gastric acid secretion potentially causing vitamin B_12_ deficiency or the inhibited subcutaneous vitamin D synthesis (75% reduction of vitamin D synthesis in people aged 65 [54] in addition to a lowered renal conversion of 25-hydroxycholecalciferol (calcifediol) to the active 1,25-dihydroxycholecalciferol (calcitriol) [55, 56]. Zhu et al. have shown, that low levels of vitamins B_6_, B_12_ and B_9_ in 1605 people between the age of 60 and 75 correlate with a low socioeconomic status [57]. Regarding deficiencies of the essential vitamins that play a key role in the functioning of the immune system, numerous studies described below, that have been conducted in many different countries with a great extent of heterogeneity among the elderly, suggest a uniform trend (Table 1). Interestingly, the most commonly observed insufficiencies throughout the majority of the investigated countries include B_9_, vitamin B_12_, and vitamin D [51, 58]. Further observed deficiencies comprise vitamin B_1_, B_2_, B_6_, C and vitamin E, as depicted in Table 1. It has been described that a well-functioning immune system depends on the availability and exogenous supply of specific micronutrients including a great variety of vitamins, certain minerals and trace elements in the context of a well-balanced diet as well as supplementation, as certain population groups like the elderly potentially require an increased demand and even a peripheral nutrient deprivation might impair immunity [58].

The immunomodulatory properties of vitamin D are becoming more evident to date and even though the remaining three fatsoluble vitamins are comparingly underrepresented concerning immunologic research questions, there is evidence pointing towards the direction that vitamin A (antiinflammatory properties) as well as vitamin E (antioxidant properties) and interestingly also vitamin K (regulatory properties for example in respiratory diseases) contribute to a wellfunctioning immune system regarding the elderly in particular. Therefore, the following review aims to investigate the effects of fatsoluble vitamins on the aging immune system focusing on their distinct functions in immunomodulation and varying health consequences concerning deficiencies in contrast to appropriate serum levels due to an adequate supply as well as supplementation.

**Table 1:** Vitamin deficiencies in the elderly

Study population	Country	Vitamin deficiency	Reference
Beneficiaries of PACAM (60–80 y/o)	Chile	- vitamin **B**_**12**_, **D**	[53]
Community-dwelling ethnically diverse older adults (≥ 60 y/o)	United Kingdom	- significant decrease of vitamin **B**_**1**_, **B**_**6**_ and **B**_**9**_ intake during 8 months follow up - almost every micronutrient below recommended daily intake	[59]
65–93 y/o	Germany	- vitamin **B**_**9**_, **B**_**12**_, **D**	[42]
Alzheimer patients	China	- vitamin **B**_**2**_, **B**_**9**_, **B**_**12**_, **D**, **E**	[60]
≥ 65 y/o	Germany	- men and women: vitamin **B**_**9**_, **D** - women: vitamin **B**_**1**_, **B**_**2**_, **B**_**12**_	[61]
≥ 65 y/o	Ireland	- vitamin **B**_**9**_, **D**, **E** - women with increasing age: vitamin **B**_**9**_, **C**, **D** - overall deficiencies: men > women	[62]
Elderly with psychological disorders (60–75 y/o)	Iran	- vitamin **D**	[63]
50–82 y/o	India	- vitamin **D**	[64]

PACAM: Programa de Alimentación Complementaria del Adulto Mayor (special program for the elderly; micronutrient-enriched meals)

## Fat-soluble vitamins

**Fig. 1:**
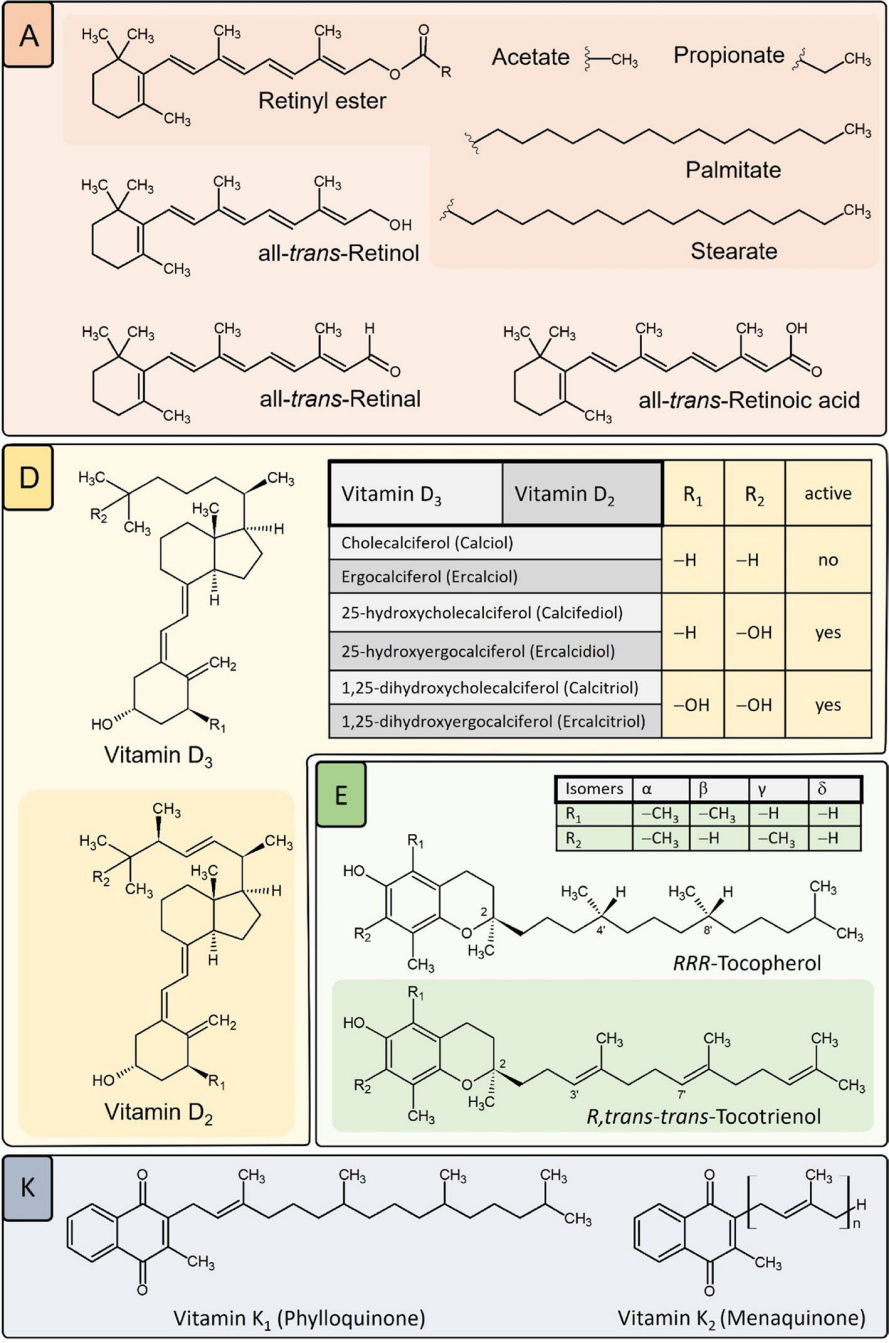
Chemical structures of fat-soluble vitamins A, D, E and K. Vitamin A comprises the retinoids, retinol, its main retinyl esters, and its later metabolites retinal and retinoic acid. The carotenes show further vitamin A activity. α-, β-, γ-carotene and the carotenoid β-cryptoxanthin can be converted to retinol (not shown). The most active forms of vitamin D are dihydroxylated cholecalciferol from skin and dihydroxylated ergocalciferol from some mushrooms and plants. Vitamin E refers to a group of 4 tocol- and 4 tocotrienol isomers (α, β, γ, δ) with α-tocopherol activity. Of the eight possible stereoisomers, only the 2R,4’R,8’R (RRR) tocopherol stereoisomers occur naturally. With the tocotrienols, only the 2R,3‘trans-7‘trans configuration is found in nature. The vitamin K vitamers comprise compounds with a quinone ring and carbon tails of different lengths and saturation status. Vitamin K_1_ has a phytyl sidechain of 20 C-atoms. The most studied vitamin K_2_ (menaquinone) derivatives have 4 and 7 isoprenoid units (menaquinone-4 (MK-4) and MK-7), respectively

Vitamins of natural or chemical origin are essential micronutrients vital for human health that perform a variety of essential body functions. With the exception of niacin which derives from tryptophan and vitamin D, which is synthesized from cholesterol, vitamins need to be consumed in trace amounts via diet or supplements, whereas their bioavailability differs greatly depending on the quality of food and interindividual factors like age, sex or physiological functions, among others [65–68]. Fat-soluble vitamins including vitamins A, D, E and K display similar structural characteristics as they resemble lipophilic compounds constructed of isoprenoid units and are of great importance due to their distinct functions regarding immunomodulation, which in turn emphasizes their part in human health or disease (Fig. 1). The absorption of fat-soluble vitamins depends on the consumption of dietary fat to facilitate their bile salt-dependent micellar solubilization, followed by the release of said vitamins to the bloodstream bound to carrier proteins like lipoproteins reaching their side of action or getting stored in the liver or fat-associated tissues [69–72].

### Vitamin A

#### Vitamin A: general characteristics and physiological function

The term vitamin A refers to natural substances containing an unsaturated alicyclic ring and encompasses all of the animal-, plant-derived or chemically synthesized retinoid-related compounds that share similar physiological functions and a structure based on an unsaturated isoprenoid chain consisting of four isoprene units and five conjugated double bonds (Fig. 1) [72–74]. Naturally occurring retinoids include retinyl esters (all-*trans-*retinyl esters) or carotenoids (tetraterpenoids) such as α-carotene, β-carotene, lutein, lycopene and cryptoxanthin along with all-*trans-*retinol (alcohol; parent compound), retinal (all-*trans-*retinaldehyde; oxidation product), all-*trans-*retinoic acid (ATRA; tretinoin), 9*cis-*retinoic acid (alitretinoin), 11-*cis-*retinaldehyde (retinal) and 13‑*cis*‑retinoic acid (isotretinoin) [73, 75].

Retinol, retinal and retinoic acid (RA) exert physiological functions with RA being the most biologically relevant. Certain carotenoids from plants are considered as provitamin A since they can be metabolically converted into the active version of vitamin A [76, 77]. Moreover, even though retinol is the most common form of retinoids found in the human body, the biologically active derivates comprise the oxidized 11-*cis-*retinal and ATRA [78, 79].

Dietary vitamin A equivalents like retinyl esters originating from animal-based products are being intestinally metabolized into retinol by triglyceride lipase or phospholipase B, associated into chylomicrons and secreted into the lymphatic system before reaching the systemic circulation and ultimately being delivered to the liver as the main storage for retinoids or to the side of action resulting in their binding to the retinol-binding protein receptor (RBPR), which enables oxidation of retinyl esters or retinol into ATRA upon entering the target cell. Utilization of stored vitamin A happens by releasing the retinols into the blood where they are already attached to the retinol-binding protein (RBP) or bind to other transport proteins such as albumin [73]. Due to differences in bioavailability it is worth mentioning that retinols and retinyl esters originating from animal products surpass plant-derived carotenoids significantly in terms of absorption [78, 80]. Carotenoids may pass unmetabolized (up to twothirds of the carotenoids like β-carotene) or be converted into retinal and subsequently oxidized into ATRA, hence biologically active versions of vitamin A (only onethird), or reduced to retinol depending on administered amount, storage levels and the amount of concomitantly consumed dietary fat [73, 81].

**Fig. 2:**
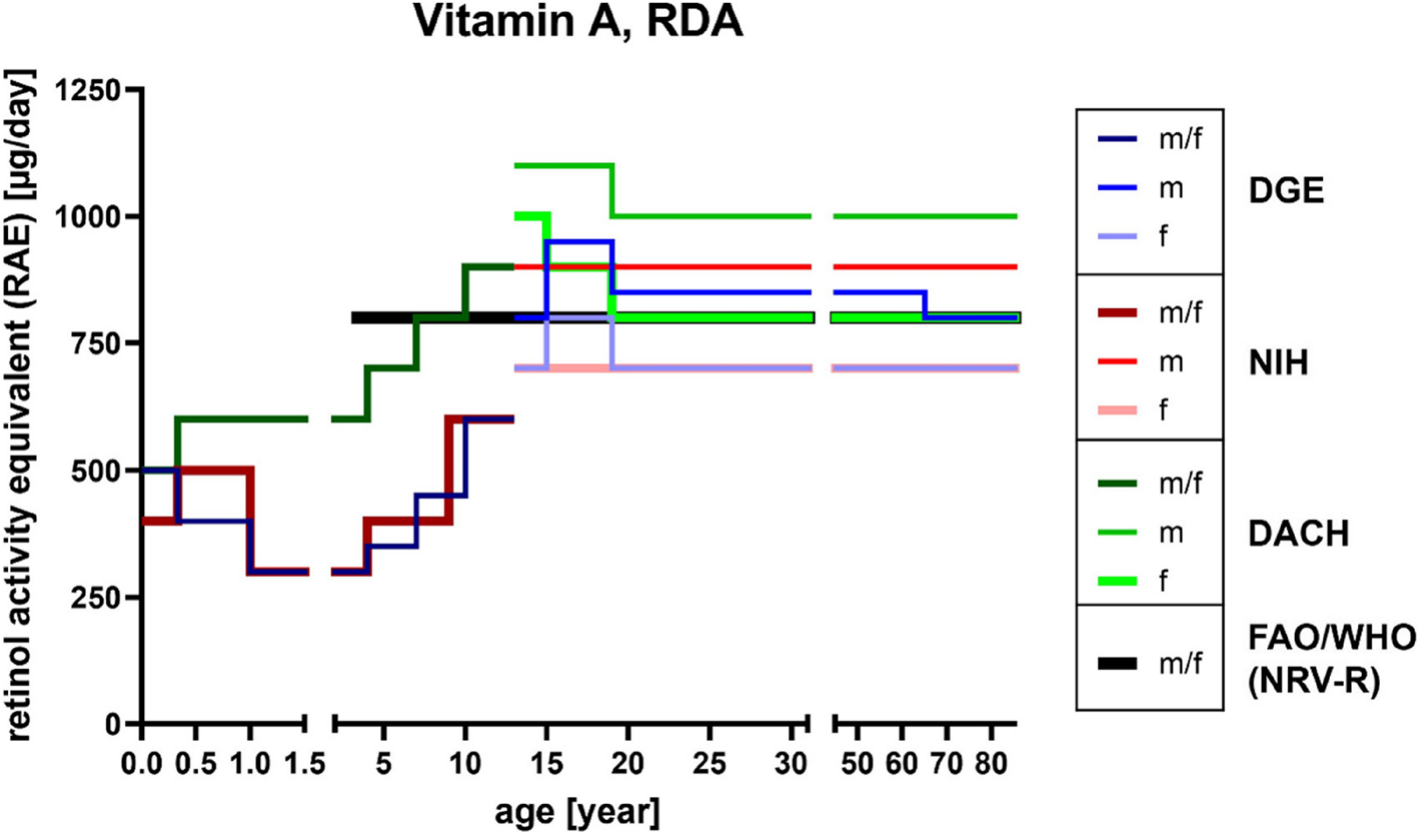
RDA reference values of vitamin A (as RAE). References according to D-A-CH [87], DGE [88], NIH [83] and the NRVs-R of FAO/WHO [86]. D-A-CH, Deutschland, Austria, Confoederatio Helvetica (eng. GSA, Germany, Switzerland, Austria); DGE, Deutsche Gesellschaft für Ernährung (eng. German Nutrition Society); FAO, Food and Agriculture Organization; NIH, National Institutes of Health; NRV-R, Nutrient Reference Value-Requirement; RAE, Retinol Activity Equivalents; RDA, Recommended Daily Allowance; WHO, World Health Organization

**Fig. 3:**
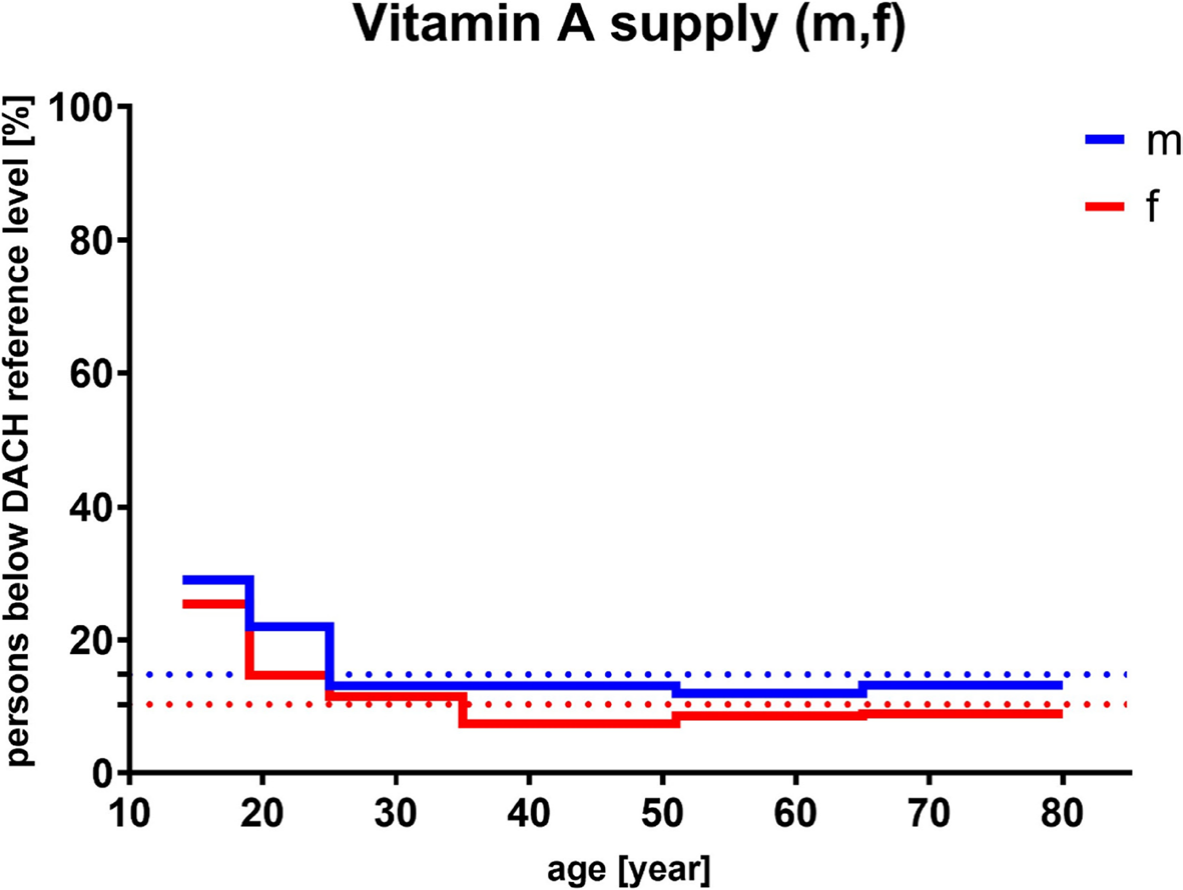
Results of the German National Nutrition Survey II (2005–2007) [89]. Percentage of people (male (m), *n* = 7093; female (f), *n* = 8278) of different ages who do not reach the recommended vitamin A (as RAE) intake according to D-A-CH reference values [87]. Mean values are represented as dotted lines. D-A-CH, Deutschland, Austria, Confoederatio Helvetica (eng. GSA, Germany, Switzerland, Austria); RAE, Retinol Activity Equivalent

Since the 1960s, Retinol Equivalents (RE) were used to describe vitamin A activity from retinol and carotenoids. However, this unit was ought to be replaced by Retinol Activity Equivalents (RAE) after it was discovered that the biological activity and hence the conversion rate of carotenoids to the metabolically active retinol is only half of what was originally estimated. As a result, RAE was introduced and partially replaced the old unit depending on the referring health institution: 1 µg RAE equals 1 µg retinol, 2 µg supplemental β-carotene, 12 µg dietary β-carotene, or 24 µg dietary α-carotene or β-cryptoxanthin, whereas 1 µg RE required only 6 µg β-carotene [82–85]. Therefore, Recommended Dietary Allowance (RDA) for vitamin A consumption amounts to up to 1000 µg of RAE for male adults and 800 µg RAE for female adults (compare age groups 50 and older; Deutschland, Austria, Confoederatio Helvetica (D-A-CH (eng. Germany, Switzerland, Austria (GSA)), Deutsche Gesellschaft für Ernährung (DGE; eng. German Nutrition Society) and National Institutes of Health (NIH) reference values; Fig. 2). These numbers are in line with the Nutrient Reference Values (NRVs) which define 800 µg RAE (or RE) as an adequate amount for contributing to an overall healthy diet concerning the general population [86]. During times of pregnancy and lactation the recommended intake increases up to 1300 µg RAE (lactating women aged 19–50 y/o). Interestingly, as shown in Fig. 3 and based on the results of the German National Nutrition Survey II (2005–2007) [61], more than 80% of the people aged 50 and older seem to be properly supplied with vitamin A which positively correlates to the data of the actual vitamin intake compared with the reference values of D-A-CH, DGE and NIH for men and women (Figure S1).

As already mentioned, animal products like most types of fish, oils, eggs, dairy products and most importantly liver contain vitamin A as retinol or retinyl esters (retinyl palmitate), whereas plants and plant-based products predominantly provide provitamin A in the form of carotenoids which are containing one or more unsubstituted β-ionone rings (α-carotene; β-carotene: dark leafy green vegetables, carrots, tomatoes; β-cryptoxanthin: citrus fruits) [73, 78, 90–92]. Concerning animal produce, the vitamin A content of the final product depends on the levels of naturally occurring or supplemented β-carotene / preformed vitamin A in the feed [93, 94]. Considering bioactivity and function of retinoids, many health claims regarding the general population as target group have been proposed and verified as such including vitamin A as a contributor to the maintenance of normal skin, mucous membranes (lung, intestines, nose, eyes), normal vision, cell specialization / differentiation and normal function of the immune system [95].

The fat-soluble vitamin exerts various effects upon consumption due to its many derivatives and their tissue- and process-specific properties [73]. Generally, biologic activity of ATRA such as gene expression or inhibition through direct DNA interaction is being exerted by binding to nuclear retinoid receptors (ligand-activated transcription factors) like (all-*trans*) retinoic acid receptors (RARs) or retinoid X receptors (RXRs) which together build a heterodimer [83, 96–99]. Out of all retinoids, only retinal is able to contribute to the process of normal vision. The conversion of light into optic perceptions being transmitted to the brain via the optic nerve happens in the rods by its association to the protein opsin consequently forming rhodopsin, before 11-*cis*-retinal gets photo-isomerized to all‑*trans*‑retinal by the absorption of light [83, 100–102]. Thus, an adequate level of vitamin A is crucial as it plays a critical role in various biological functions such as vision, growth, hematopoiesis and, in light of this review article, it exerts immunomodulatory as well as antioxidant activity [72, 103].

#### Vitamin A: modulatory effects on the aging immune system

As already mentioned, vitamin A exerts various effects on the innate as well as adaptive immune system, as it is involved in DC-, T helper (Th) cell- and cytotoxic T cell-maturation along with an enhancing effect on lymphocyte activity and antibody production [72]. Vitamin A plays a role in regeneration of mucous tissue and skin which in turn enhances the barrier against invading pathogens [104, 105]. RA inhibits the development of Th1 cells while promoting Th2 development (humoral Th2 cell response via antigen-presenting cells; increase in Th2 associated transcription factors; modulation of cytokine secretion), thereby mediating Th cell balance. In line with that, RA mediates maturation and antigen presentation of DCs, whereas a deficiency in vitamin A causes an increase in inflammation caused by macrophage-mediated IL-12 and interferon-γ (IFN-γ) production [106–110].

In relation to adaptive immunity, vitamin A causes an increase in IL-2 levels, triggering the proliferation and differentiation of T cells into regulatory T cells (T_regs_), which in turn plays a crucial role in preventing autoimmune disorders [111, 112]. Furthermore, 13-cis-RA has been shown to increase the overall amount of peripheral blood lymphoid cells that express surface markers for Th cells, whereas β-carotene had an impact on the percentage of cells expressing NK cell markers. Both compounds resulted in a slight increase in cells expressing transferrin- and IL-2 receptors [113].

Contrary to the immune-boosting effects of vitamin A, an observational prospective cohort study found that there appears to be no significant connection between differing micronutrient levels (vitamin A among others; no detectable insufficiency in any participant) and the serologic response to influenza vaccination measured by hemagglutination inhibition (HAI) titer in 205 community-dwelling adults aged 65 and older, which contradicts the assumption that decreased levels of said micronutrients would be causing decreased HAI responses to vaccination [114]. These observations are supported by the findings of Gardner et al. [115], which investigated the immune responses and plasma micronutrient levels (β-carotene and retinol among others) in 61 healthy elderly (mean 81 y/o) compared to 27 young (mean 27 y/o) participants before and after influenza vaccination. The elderly showed comparingly low influenza vaccine induced proliferation and IFN-γ levels, as well as lower post-vaccination antibody titers, but these differences seemed to be independent from differing micronutrient levels [115].

A potential explanation for the observed immunologic differences in the elderly vs. young might be physiological senescence. In accordance with this concept, a study investigating age-related changes in RAR and RXR subtypes gene expression and tissue transglutaminase activity in human peripheral blood mononuclear cells (PBMCs) before and after supplementing 13-*cis*-retinoic acid found that the expression of RXR-β in healthy elderly men (65.4 ± 3.8 y/o) was significantly reduced compared to younger men (26.1 ± 4.1 y/o) [116]. In line with that, Farges et al. [117] also investigated the impaired immune response with age in relation to carotenoid intake (β-carotene, lycopene, lutein), finding that age-related changes in immune markers such as higher serum IgA levels and altered lymphocyte subpopulations (increase in memory Th cells (CD4 + CD45RO+) and natural killer (NK) cells along with a decrease in naïve Th cells (CD4 + CD45RA+) and B lymphocytes) and impaired neutrophil activity, which could be modulated by carotenoid intake, depended only marginally on dietary carotenoid depletion and repletion [117].

Moreover, a study conducted by Minet-Quinard et al. [118] found that oral supplementation with 13-*cis*-RA neither had an impact on the composition of leukocyte subpopulations, nor the functions of PBMCs (IL-2 production, membrane expression of CD25), whereas certain functions of polymorphonuclear cells, namely adhesion and migration, were affected. No differences in age could be observed as the immune responses in young participants (25 ± 4 y/o) are comparable to those of the healthy elderly (65 ± 4 y/o) [118]. In accordance, supplementation with vitamin A could not reduce the occurrence of antibiotic-treated bacterial infections among elderly nursing-home residents, as demonstrated by a double-masked, placebo-controlled trial [119].

While vitamin A intake of healthy study participants seems to exert only little effects on the immune system, studies have proven the relationship between retinoids and a positive disease outcome. In light of the recent global COVID-19 pandemic, Al-Saleh et al. [120] measured serum levels of various trace elements, vitamins and antioxidant enzyme activities in COVID-19 patients in correlation to disease severity. 37% of the patients were deficient in vitamin A and a 23% decrease in serum levels could be observed in patients having severe symptoms, which diminished after the adjustment for inflammatory markers pointing towards the fact that inflammation might play a role in altering the relationship between serum vitamin A and disease severity [120].

These findings are supported by another prospective, multicenter observational cross-sectional study conducted in 2021, analyzing plasma levels of vitamin A in patients having severe acute respiratory syndrome coronavirus type 2 (SARS-CoV-2). There has been a significant correlation between reduced vitamin A levels and inflammation (C-reactive protein (CRP), ferritin) along with COVID-markers (reduced lymphocyte count, lactate dehydrogenase (LDH)). In general, disease severity and mortality correlated significantly with lower plasma vitamin A levels [121]. In a study cohort with patients suffering from common variable immunodeficiency (CVID) which results in a reduced antibody production and recurrent bacterial infections, hence increased susceptibility to infections, plasma vitamin A levels were decreased compared to healthy controls and supplementation resulted in decreased levels of TNF-α and increased levels of IL-10. However, it remains uncertain whether vitamin A deficiency is a cause or a consequence of the infection. Evidence points into both directions [122, 123].

Moreover, higher IgA levels and phytohemagglutinin (PHA)-stimulated PBMC proliferation after supplementation has been observed in vivo [124]. These findings are supported by a similar study conducted in 2013, also investigating the role of vitamin A in CVID. ATRA has been shown to restore defective immune responses in CVID-derived B cells as it improved proliferation and IL-10 secretion among others [125]. Regarding vitamin A as a potential treatment for atherosclerosis by influencing forkhead box protein 3 (FoxP3) and transforming growth factor (TGF)-β expression, Mottaghi et al. [126] administered retinyl palmitate or placebo to atherosclerotic patients and a healthy control group, before studying the gene expression of T_regs_. The authors conclude that vitamin A impacts the expression of T_regs_ and in turn their suppressing actions on effector T cells. Therefore, supplementation might play a role in progression of atherosclerosis [126]. Furthermore, immune stimulative effects of vitamin A (retinyl palmitate or 13cisRA) such as an increased lymphocyte blastogenesis response to PHA in patients with metastatic unresectable squamous cell carcinoma of the lung could be observed as well as an overall immune potentiating effect, making it suitable for the application in combination therapies [127]. A summary of the aforementioned study outcomes is provided in Table S1.

As depicted, supplementation of vitamin A has its benefits concerning healthy elderly as well patients suffering from certain diseases, whereupon the effects in healthy elderly seem less pronounced. Hypervitaminosis and toxicity often occur in the context of supplemental misuse or high consumption of food containing lots of preformed vitamin A such as liver or eggs, causing rather mild symptoms such as loose stools, headache, nausea and vomiting, but these side effects occur fairly rarely and usually stop in close proximity to discontinuation [73, 85, 92].

In contrast, inadequate levels of vitamin A might result in the commonly observed phenomenon night blindness or impaired vision due to malfunctioning of the rods in dim light (retina) and can lead to hyper keratinization of the eye epithelial tissue and ultimately to blindness if the deficiency is severe and long-term [83, 101, 128]. Regarding respiratory symptoms, a vitamin A deficiency manifests in epithelial modifications leaving the person prone to bacterial and viral invasion and an overall higher susceptibility to inflammatory processes and infections [73, 129]. Oral administration of retinol equivalents is common practice in the food and food supplement industry whereupon vitamin A usually comes in the form of retinyl acetate, retinyl palmitate or β-carotene with absorption rates ranging between 70 and 90% (preformed vitamin A esters) and 8.7–65% (β-carotene), respectively [130–132]. The amount of orally administered retinol equivalents varies greatly, but commonly comprise 3000 µg RAE given alone, or 750–1050 µg RAE in multivitamin supplements [133]. Figure S2 provides a summary of the actual intake of vitamin A supplements compared with the reference values of D-A-CH, DGE and NIH for men and women, based on the results of the German National Nutrition Survey II.

### Vitamin D

#### Vitamin D: general characteristics and physiological function

Vitamin D comprises the following endogenous and synthetic compounds. D_1_ (1 + 1 mixture of ergocalciferol and lumisterol), D_2_ (ergocalciferol), D_3_ (cholecalciferol), D_4_ (22,23-dihydroergocalciferol) and D_5_ (sitocalciferol) [134]. The main pre-forms of active vitamin D in the body are ergocalciferol and cholecalciferol whereby the latter is of primary importance and supposedly more efficacious than ergocalciferol (Fig. 1) [135]. Food sources of cholecalciferol are seafood including fatty fish such as trout, salmon, mackerel and herring, but it is also found in egg yolks [135].

Activation of cholecalciferol occurs by hydroxylation in the liver to become 25-hydroxyvitamin D (calcifediol) and another hydroxylation in the kidneys to be converted into the biologically active 1,25-dihydroxyvitamin D (calcitriol) [136, 137]. Cholecalciferol exhibits a steroid-like structure and can be physiologically synthesized from 7-dehydrocholesterol in the skin after exposure to ultraviolet B (UVB)-light (wavelength: 290–315 nm and a dosage value of at least 18 mJ/cm²) [135, 138, 139]. Many countries are located in latitudes where sunlight is sometimes insufficient to enable cholecalciferol production in the skin [135, 140]. Therefore in Nordic countries, dairy products are often fortified with cholecalciferol [135]. Due to the endogenous synthesis in the context of UVB-light exposure, cholecalciferol can be considered a prohormone rather than a vitamin. Consequently, calcitriol is regarded as a steroid hormone and reacts with the associated vitamin D receptor (VDR) in a similar way to other steroid hormones with their respective intracellular receptors [141].

The hydroxylation of cholecalciferol in the liver is catalyzed by cytochrome P450 vitamin D 25-hydroxylases (for example CYP2R1, CYP2D11 and CYP2D25) yielding calcifediol [136, 137]. Calcifediol is the main form of vitamin D in plasma and, at the same time, the storage form enabling it as an indicator for assessing the vitamin D status in blood samples in contrast to calcitriol with its short plasma half-life of 4–8 h [135, 137, 142, 143]. Vitamin D binding proteins (VDBP) are responsible for the transport of calcifediol to the kidneys, where the final hydroxylation takes place [137]. In the proximal tubule, physiologically active calcitriol is synthesized by 1-α-hydroxylase (CYP27B1) [137].

Calcitriol regulates calcium and phosphate levels by promoting calcium absorption in the gastrointestinal (GI) tract and stimulating reabsorption of calcium and phosphate by the kidneys [137, 144, 145]. In turn, calcitriol deficiency correlates with calcium deficit and is additionally an important regulator of sex hormone levels [137, 144]. High phosphate levels suppress the conversion of calcifediol into calcitriol [137, 145].

Regarding the bioactivity of vitamin D, many health claims for the general population have been authorized in the European Union [95]. Vitamin D contributes to the normal absorption and utilization of calcium and phosphorus, to the maintenance of normal teeth and muscle function and it is further needed for normal growth and development of bones in adults and children. In addition, vitamin D helps to reduce the risk of falling associated with postural instability and muscle weakness (risk factor over 60 y/o). It also contributes to the normal function of the immune system in adults and children and has a role in cell division. Together with calcium, it helps to reduce the loss of bone mineral in post-menopausal women [95].

More than 36 cell types possess the VDR and interestingly paracrine production of calcitriol occurs in more than ten extrarenal organs [141]. The effects of calcitriol are remarkably diverse involving the regulation of more than 1000 genes important for a wide variety of cells and tissues [137, 146]. To acquire comprehensive data on vitamin D signaling, Dimitrov and colleagues analyzed raw data from 94 gene expression profiles (80 from humans, 14 from mice) regulated by calcitriol or its analogs [147]. Several of the identified genes are also involved in biochemical pathways of cancer cells [137] and regulate immune responses, cell proliferation, differentiation and apoptosis [137, 146, 148]. Calcitriol is also involved in insulin secretion by the pancreatic β-cells, maintenance of heart function, blood pressure regulation as well as brain and fetal development [141]. Vitamin D even modulates the composition of the GI microbiome [149].

**Fig. 4:**
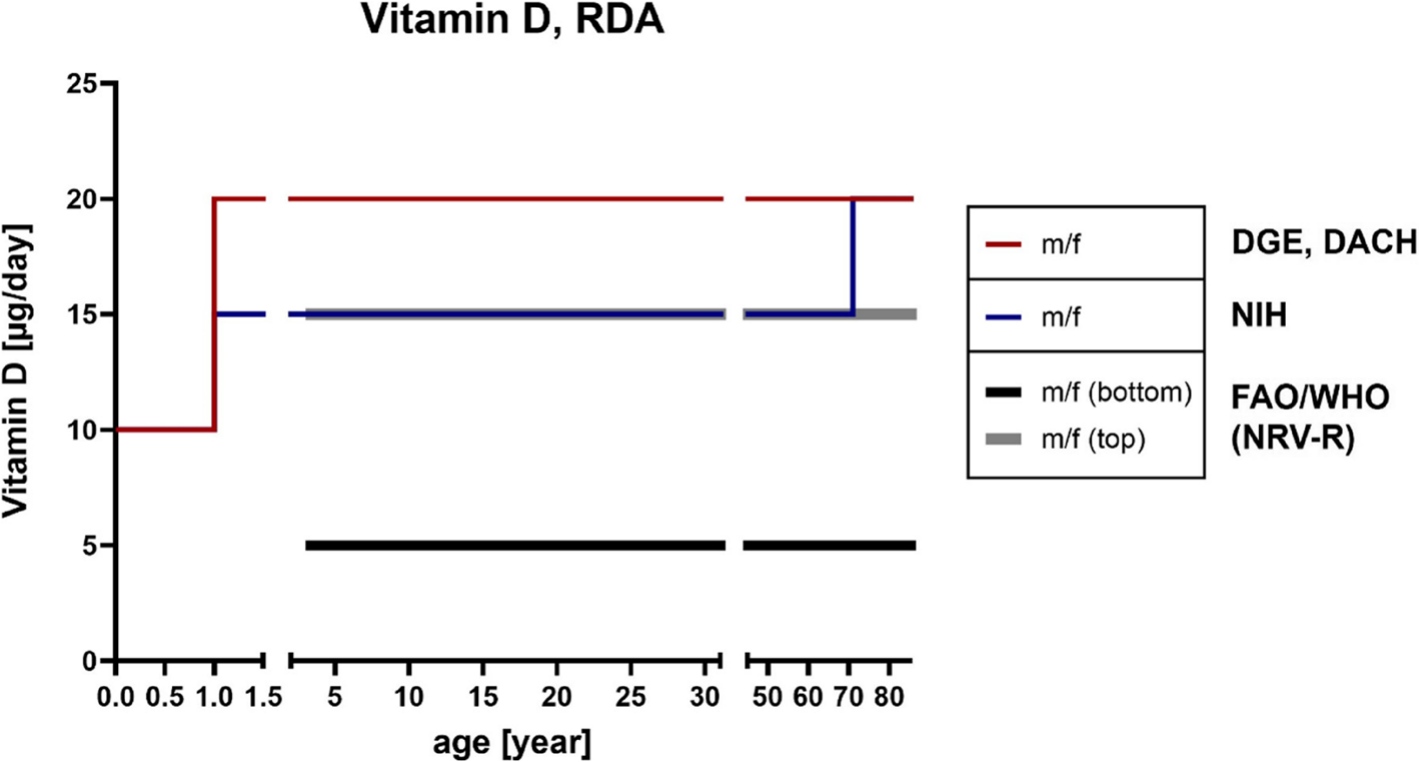
RDA reference values of vitamin D. References according to D-A-CH [87], DGE [156], NIH [157] and the NRVs-R of FAO/WHO [86]. D-A-CH, Deutschland, Austria, Confoederatio Helvetica (eng. GSA, Germany, Switzerland, Austria); DGE, Deutsche Gesellschaft für Ernährung (eng. German Nutrition Society); FAO, Food and Agriculture Organization; NIH, National Institutes of Health; NRV-R, Nutrient Reference Value-Requirement; RDA, Recommended Daily Allowance; WHO, World Health Organization

**Fig. 5:**
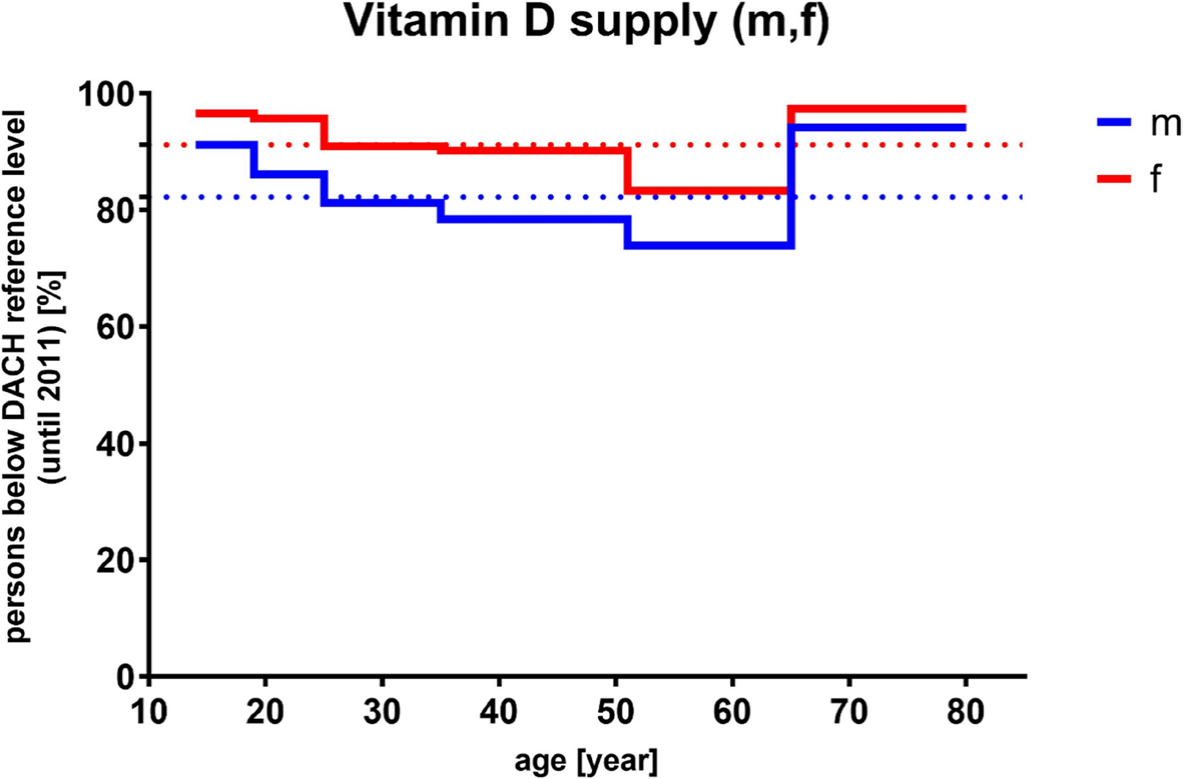
Results of the German National Nutrition Survey II (2005–2007) [155]. Percentage of people (male (m), *n* = 7093; female (f), *n* = 8278) of different ages who do not reach the recommended vitamin D intake according to D-A-CH reference values (valid until 2011). Mean values are represented as dotted lines. D-A-CH, Deutschland, Austria, Confoederatio Helvetica (eng. GSA, Germany, Switzerland, Austria)

Accordingly, there is a big variety of diseases in the context of vitamin D insufficiency or deficiency and, for example, about two-thirds of the world’s population seemingly do not get enough vitamin D for the maintenance of an optimal bone density [141, 150]. In this respect, 20 ng/mL (50 nmol/L) calcifediol in plasma corresponds to a sufficient level whereas less than 10–12 ng/mL (25–30 nmol/L) indicates deficiency [135]. However, for optimal health conditions, there is evidence pointing towards the direction that higher serum concentrations might be beneficial [151, 152]. The RDA for vitamin D consumption amounts to up to 20 µg/day (compare age groups 50 and older; DACH, DGE and NIH reference values; Fig. 4) whereas correlating NRVs define 5–15 µg vitamin D to be enough for ensuring an adequate supply concerning the general population [86]. Moreover, a sufficiently high magnesium intake appears to reduce the risk of vitamin D deficiency [153], while the intake of various medications, e.g., metformin, loop diuretics, statins, antidepressants or certain chemotherapeutic agents among others can alter vitamin D status [154]. Interestingly, as shown in Fig. 5 and based on the results of the German National Nutrition Survey II (2005–2007) [155], approximately 80–100% of the people aged 50 and older do not each daily intake recommendations, which matches the data of the actual vitamin intake compared with the reference values of DACH, DGE and NIH for men and women (Figure S1).

#### Vitamin D: modulatory effects on the aging immune system

Among numerous pleiotropic physiological effects, calcitriol and its structural analogues are known to play a downright important role concerning immune reactions which have been described in an epidemiologic context correlating low vitamin D status and the occurrence of autoimmune as well as inflammatory diseases including, for example, Hashimoto thyroiditis, multiple sclerosis or inflammatory bowel disease, with higher prevalences in northern countries with decreased vitamin D synthesis [147, 158–160]. Along with these observations, calcitriol is known of influencing immunity regarding anti-inflammatory responses, tolerogenic actions and the prevention of immune overstimulation [68]. Regarding allergic reactions, vitamin D deficiency (93% of patients) have been associated with an increased severity of allergic rhinitis and accordingly elevated levels of IgE [161].

Moreover, the fatsoluble vitamin influences the human microbiota by retaining intestinal homeostasis accomplished due to an increased cellular production of antimicrobial proteins from, for example macrophages and monocytes through RXR/VDR-signaling induction of promotor sequences of cathelicidin antimicrobial peptide etc., thereby preventing intestinal bacterial translocation and, on a large scale, the development of auto-inflammatory / metabolic dysfunctions, along with its role in maintaining intestinal epithelial cell integrity (production of nitric oxide due to upregulation of endothelial nitric oxide synthase (eNOS)), which in turn minimizes epithelial damage caused by bacterial lipopolysaccharides (LPS) [151, 162–168].

In contrast, it must be noted that microbiota composition impacts the hydroxylation of calcifediol by CYP27B1, as shown in germ-free mice exhibiting high fibroblast growth factor 23 (FGF23)-levels which correlate to decreased cholecalciferol-metabolization rates [169]. Generally speaking, vitamin D’s immunogenic capacity heavily relies on the expression of VDRs in the majority of immune cells encompassing DCs differentiation of T_regs_, monocytes, macrophages, Th cells (induction of Th2 cells and associated IL-3, IL-4, IL-5 and IL-10; reduction of pro-inflammatory Th1 and related IL-2, IFN-γ and TNF-α, reduction of Th9 and Th22 cells; promotion of a tolerogenic rather than pro-inflammatory environment), T- and B cells among others, as well as their ability of expressing the enzyme 25-(OH)-D-1α-hydroxylase (CYP27B1 allele), thus influencing innate and adaptive immunity, including B-cell-mediated humoral defense [108, 151, 170–175].

In the context of immune tolerance, tolerogenic DCs have been gaining much attention with regard to the fact that calcitriol has been shown to promote antigen-presenting cells (APCs) to increase their tolerogenic properties in addition to the reduction of major histocompatibility complex (MHC) class II expression [151, 176–178]. The treatment of myeloid DCs (tolerogenic and immunogenic features) with calcitriol induced the upregulation of CC-chemokine ligand (CCL) 22 production (a chemokine that attracts T_regs_) but decreased CCL17 production, whereas a reduction of IL-12p75 production, an increase in CD4^+^ suppressor T cell activity and an altered DC-mediated Th1-lymphocyte-development capacity via cytokine secretion could be observed. Interestingly, similar effects could not be noted upon vitamin D treatment of blood plasmacytoid DCs (tolerogenic features) [179]. Similarly, calcitriol exposure led to the induction of (differentiating) monocyte-derived tolerogenic DCs due to metabolic reprogramming [180].

Moreover, calcitriol has been described to influence cell-specific regulatory processes including certain aspects of the NOD-like pattern recognition receptor signaling, along with enhancing the catabolism of branchedchain amino acids (BCAAs) in monocytic cells potentially resulting in the suppression of BCAA-regulated mTOR signaling [147]. Genomic ramifications by calcitriol are being caused by the RXR/VDR nuclear receptor heterodimer complex which attaches to vitamin D response elements (VDREs) thereby serving as a transcription factor inducing gene expression through targeting calcitriol related promotor sequences [181–184]. Interestingly, with regard to vitamin D and clinical endpoints (anti-tumoral effects; B cell lymphoma), calcitriol has been shown to suppress the proliferation of immune cells and production of IFN-γ, thereby altering the efficacy of rituximab (therapeutic antibody)-mediated antibody-dependent cell-mediated cytotoxicity (ADCC) on effector γδ-T cells along with enhancing the cytotoxic effects of effector NK cells in vitro [185].

Regarding our aging society, certain aspects in terms of immunomodulatory changes have to be highlighted. A study on healthy controls described an inverse correlation between age (especially those older than 60 y/o), concomitantly low levels of circulating calcifediol and altered innate immune markers being the expression and function of various TLRs on immune cells, particularly those related to viral responses, and low levels of cathelicidin, which in turn were positively associated with calcifediol levels [186]. Along with that, decreased serum calcifediol (manifested hypovitaminosis D) has been shown to reinforce symptom severity / mortality and deteriorate disease outcome of patients with acute respiratory failure due to COVID-19 [187], as well as increase the risk of incident hospitalized pneumonia in generally healthy study participants aged (53–73 y/o) [188]. In accordance with Castillo et al., COVID-19 patients might benefit from calcifediol supplementation [189].

In contrast, no significant improvement of IL-1β, IL-6, IL-10, TNF-α, IL-4, IL-12p70, IL-17 A, IFN-γ, granulocyte-macrophage colony-stimulating factor (GM-CSF), IL-8, IFN-inducible protein-10 (IP-10), macrophage inflammatory protein-1β (MIP-1β), monocyte chemoattractant protein-1 (MCP-1), vascular endothelial growth factor (VEGF), and leukocyte count could be observed following the oral application of a single high dose of 200,000 IU cholecalciferol in hospitalized patients with moderate to severe COVID-19 (55.5 ± 14.3 y/o) [190]. Similar outcomes were reported by Barnes et al. [191]. Even though daily supplementation of 15 µg cholecalciferol significantly increased calcifediol serum concentrations in the elderly (mean 64 y/o), no explicit impact on cytokine concentration (CRP, IL-6, IL-10, soluble CD40 ligand, TGF-β, TNF-α and fibrinogen) could be observed [191]. Interestingly, a study conducted on Mexican healthy elderly (decreased age-associated endogenous vitamin D synthesis) suggested that despite living in a country with appropriate UV radiation exposure due to lower latitude, vitamin D insufficiency (91.3%) is a ubiquitous matter throughout the year resulting in correlating TNF-α serum levels, potentially explaining the increased susceptibility of older adults to systemic inflammation and associated diseases [192].

Subsequently, a study investigating gene polymorphisms in the context of genetic susceptibility for vitamin D deficiency and COVID-19 severity might shed some light on the topic, with an emphasis on the lack of personalized approaches for viral infections due to interindividual differences [193]. Freitas and colleagues (2021) showed that among others, polymorphisms in the vitamin D-binding protein (encoded by GC gene) correlate to disease severity in Portuguese, hospitalized patients. In close proximity to the findings of Elizondo-Montemayor et al. [192] but under altered premises, Australian elderly (60–84 y/o), supposedly representing a population group with low incidences of vitamin D deficiency, consumed 60,000 IU cholecalciferol on a monthly basis for five years, but no significant effects on hospitalization due to infection, despite a decline in the number of extended hospitalizations (over 6 d), could be observed, suggesting that general supplementation in people with adequate vitamin D serum concentrations exert only little effects, whereas its role in infectious disease is reinforced [194].

An inverse relationship between elevated serum calcifediol and CRP being a marker of systemic inflammation could be observed [195]. Concerning the impact of cholecalciferol supplementation (100,000 IU/15 d for three months) on influenza vaccine response (seroprotection and immune response), no significant changes in cathelicidin levels, antibody titer and ROS production in contrast to elevated TGF-β plasma levels could be observed in elderly study participants with suboptimal vitamin D levels (< 30 ng/mL / 75 nmol/L) 28 days after vaccination [196].

Interestingly, weekly supplementation of 20,000 IU cholecalciferol over the course of three to five years resulted in transcriptomic changes encompassing the expression of various vitamin D-regulated genes involved in the IL-signaling pathway, apoptosis signaling pathway, oxidative stress response and gonadotropin-releasing hormone receptor pathway after stratifying for subjects with the lowest or highest serum calcifediol levels [197]. A summary of the aforementioned study outcomes is provided in Table S1.

In light of aging-associated changes such as reduced dietary bioavailability (gallbladder removal or gastrointestinal diseases), decreased amounts of 7-dehydrocholesterol in the skin and concomitantly the inhibited conversion rates of ergocalciferol into cholecalciferol (decrease by factor 3 compared to young) as well as the commonly observed vitamin D deficiency (calcifediol < 20 ng/mL / 50 nmol/L [198]; calcifediol < 12 ng/mL / 30 nmol/L [199]) in the elderly, representing one of the many risk groups, oral application certainly has its benefits and daily supplementation may be recommended based on scientific and epidemiologic evidence, especially since excessive exposure to UV-radiation correlates to the development of skin cancer [200–203].

Since roughly 40% (6%) of the adults display an insufficient (deficient) vitamin D nutritional status due to interindividual differences referring absorption efficiency (55–99%), or altered bioavailability as a result of varying dietary lipid composition, just to name a few, general recommendations for the prevention of deficiency include daily application of 600–2000 IU cholecalciferol depending on individual factors like sunlight exposure, nutritional intake etc., whereas a linear relationship between the intake (lower dosages between 1000 and 2000 IU / 25–50 µg; extenuated effect with higher dosages) and serum calcifediol might be observed [157, 198, 199, 204–211].

However, correlating vitamin D intake via food or pharmaceuticals and actual serum concentration might entail difficulties and lead to false assumptions, because epidemiologic vitamin D status calculations based on dietary surveys barely take endogenous synthesis into account as well as differing bioavailability resulting from different food sources (for example animal products) and forms of vitamin D (ergocalciferol vs. cholecalciferol) [48, 212]. Deficiency symptoms in adults might include the development of osteomalacia (dental abnormalities, hypocalcemic seizures, bone deformities and pain) [213, 214], whereas vitamin D intoxication due to manufacturing errors or irresponsible excessive consumption (levels above 150 ng/mL (374 nmol/L) potentially leads to hypercalcemia and concomitantly vomiting, nausea, polyuria, neuropsychiatric disturbances, pain and kidney stones, or hypercalciuria, renal failure, calcification of soft tissues including coronary vessels and cardiac arrhythmias, respectively [215–218].

Nonetheless, as depicted above, moderate supplementation according to one’s personal needs implicates potential health benefits and is thought to play an important role in healthy aging. Higher supplementary absorption rates might be achieved through micellization, liposome formation or microencapsulation of vitamin D, regarding varying efficiency rates (microencapsulation more than micellization) [219]. However, as already depicted above, it has to be noted that the supplementation effects downrightly depend on interindividual differences. For that matter, Žmitek and colleagues described a positive correlation between a normal body weight (BMI < 25), lower baseline calcifediol levels representing insufficiency and ultimately supplementation efficiency [220].

In general, vitamin D from commercially available oral supplements either come in the form of ergocalciferol or cholecalciferol, whereat ergocalciferol is generated via UV-irradiation of ergosterol from yeast and cholecalciferol gets manufactured by UV-irradiation of 7-dehydrocholesterol originating from lanolin (sheep wool) or lichen [215, 221–223]. In addition, supplemented calcifediol is thought of being three to five times as effective as cholecalciferol concerning bioactivity [224, 225]. Figure S2 provides a summary of the actual intake of vitamin D supplements compared with the reference values of D-A-CH, DGE, and NIH for men and women, based on the results of the German National Nutrition Survey II. However, it must be taken into consideration that vitamin D supplementation might interact with certain medication including statins [226], corticosteroids [227–231, 229] and thiazide diuretics [230, 226].

### Vitamin E

#### Vitamin E: general characteristics and physiological function

The term vitamin E resembles lipophilic vitamers comprising tocopherols and tocotrienols (short: tocols) with a similar structure based on a chromanol ring and a 13-carbon saturated phytol sidechain (tocopherol; up to eight stereoisomers) / unsaturated isoprenoid tail (tocotrienol; two stereoisomers), which occur naturally as four homologues (α, β, γ, δ) differing in number and position of methyl groups attached to the chromanol structure, whereas α-tocopherol (αT) generally represents the biologically active form meeting the human requirements for vitamin E intake (Fig. 1) [232–237]. Dietary vitamin E esters are enzymatically hydrolyzed into non-esterified versions before being metabolized by the intestine and liver during absorption in the small intestine [238–241].

Since vitamin E does not have a distinct plasma transport protein, it is secreted into the lymphatic system by enterocytes after its association to chylomicrons and subsequently reaches the systemic circulation by passing the thoracic duct before being transferred to highdensity lipoproteins (HDL). They serve as a starting point for their distribution to all circulating lipoproteins and target tissues (for example liver), whereby the chylomicrons are degraded into chylomicron remnants [242, 243]. In the liver, the cytosolic α-tocopherol transfer protein (α-TTP) binds αT and induces its transport and incorporation into the plasma membrane [244–246]. Moreover, hepatic secretion of αT requires the involvement of the membrane protein ATP binding cassette subfamily A member 1 (ABCA1) as a mediator for assembling αT into lipoproteins for them to be delivered to extrahepatic tissues [241].

**Fig. 6:**
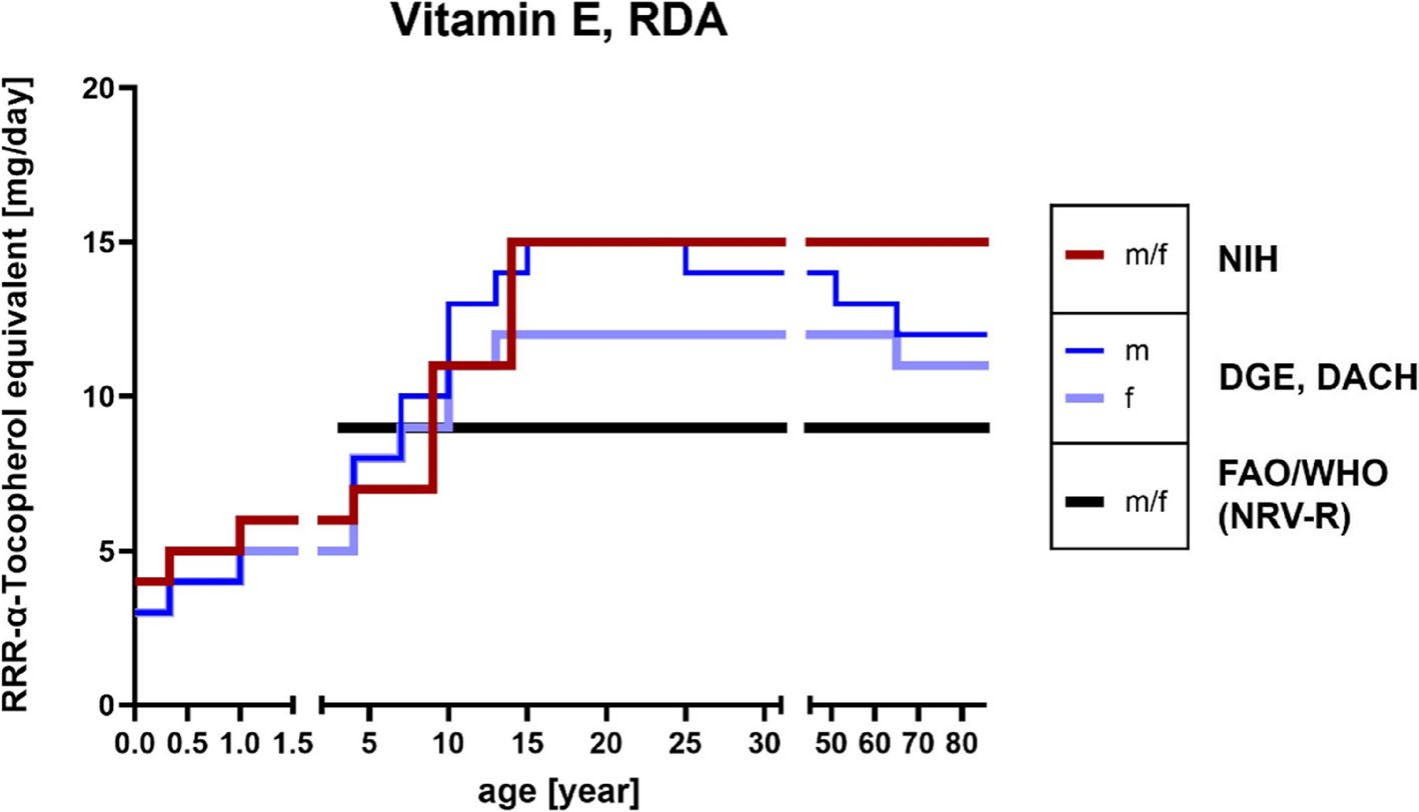
RDA reference values of vitamin E (as α-tocopherol equivalents). References according to D-A-CH [87], DGE [253], NIH [251] and the NRVs-R of FAO/WHO [86]. D-A-CH, Deutschland, Austria, Confoederatio Helvetica (eng. GSA, Germany, Switzerland, Austria); DGE, Deutsche Gesellschaft für Ernährung (eng. German Nutrition Society); FAO, Food and Agriculture Organization; NIH, National Institutes of Health; NRV-R, Nutrient Reference Value-Requirement; RDA, Recommended Daily Allowance; WHO, World Health Organization

**Fig. 7:**
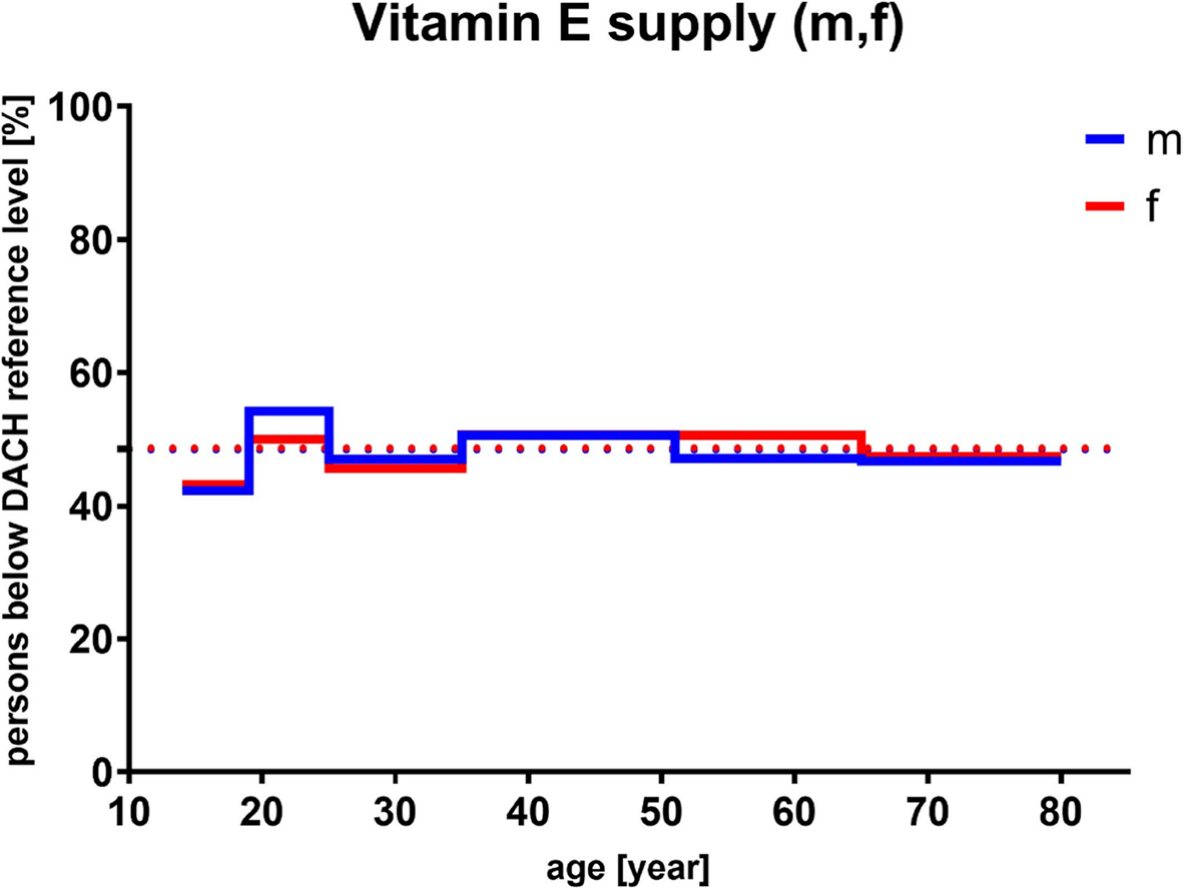
Results of the German National Nutrition Survey II (2005–2007) [252]. Percentage of people (male (m), *n* = 7093; female (f), *n* = 8278) of different ages who do not reach the recommended vitamin E (as α-tocopherol equivalents) intake according to D-A-CH reference values [87]. Mean values are represented as dotted lines. D-A-CH, Deutschland, Austria, Confoederatio Helvetica (eng. GSA, Germany, Switzerland, Austria)

Along with other food constituents, for example RA, that compete with vitamin E uptake and thereby reducing it, interindividual differences regarding absorption and bioavailability such as diseases, age, gender, lifestyle factors, interfering pharmaceuticals and genetic polymorphisms among others add up to 20–80% [243, 247–250]. Nonetheless, NRVs for vitamin E consumption equal 9 mg α-tocopherol equivalents (αTE) per day [86] and RDA amounts to up to 15 mg of αTE (compare age groups 50 and older; D-A-CH, DGE and NIH reference values; Fig. 6) which corresponds to 22.4 IU of natural (*RRR*-αT; *d*-αT) or 33.3 IU synthetic (*all rac*-αT; *dl*-αT) αT [133, 251]. Interestingly, as shown in Fig. 7 and based on the results of the German National Nutrition Survey II (2005–2007) [252], approximately half of the people aged 50 and older do not reach the daily intake recommendations, which matches the data of the actual vitamin intake compared with the reference values of D-A-CH, DGE and NIH for men and women (Figure S1). Recommendations vary greatly depending on validation method (reference markers) used, but as a rule of thumb, the intake of vitamin E should be in correlation with the amount of dietary PUFAs (1 g diene fatty acid : 0.5 mg *RRR*-αT) [243].

Natural sources of the fat-soluble compound (free forms as well as esterified with, for example, fatty acids) comprise mainly plants such as nuts (αT: almonds and hazelnuts; γT: walnuts), seeds (sesame, quinoa, pumpkin, nigella), grains / cereals (tocotrienols: barley and wheat germ) or vegetable oils (αT: sunflower oil; γT: palm oil; tocotrienols: coconut oil) and to a lesser extent animal-based products like dairy products or milk, whereas the given concentration depends greatly on external factors like growing conditions, harvest and processing [254–260].

Concerning the bioactivity and function of tocopherols / tocotrienols, certain health claims regarding the general population as target population have been proposed and verified as such including vitamin E as a contributor to the protection of cells against oxidative damage and thereby its role as a potent chain-breaking antioxidant [95]. Due to its lipophilic attributes, vitamin E derivates can accumulate in fat depots and lipid-rich regions (for example membrane of mitochondria), where they exert various effects upon consumption [261]. As already mentioned, the primary function of tocols is scavenging ROS through transferring hydrogen onto a free radical creating a non-radical product and a vitamin E radical which in turn reacts with other radicals forming a relatively stable compound and thereby preventing lipid peroxidation or platelet coagulation, which plays a role in the prevention and treatment of cardiovascular diseases (inhibition of lowdensity lipoprotein (LDL) cholesterol oxidation) [235, 262]. There have been reports of vitamin E treatment against stress being superior to vitamins A and C due to elevated levels of glutathione and catalase in contrast to reduced lipid peroxidation [263]. Apart from that, vitamin E might act synergistically with water-soluble antioxidants like phenolic acids, enhancing its effects [264, 265]. It protects membrane associated PUFAs from oxidation and participates in signal transduction [236].

Even though the panel of the European Food Safety Authority (EFSA) concluded that based on provided evidence, there has not been an established cause and effect relationship for vitamin E acting as a contributor to the maintenance of a normal immune system function regarding the absence of immune defects in participants deficient in vitamin E and the insufficient restoration of a compromised immune system after supplementation [266], there is still valid evidence pointing in the direction that the vitamin plays a role in immunity, one example being that tocopherols accumulate in the membrane of immune cells, thereby protecting their membrane integrity, proliferation and maturation as well as PUFAs along with anti-inflammatory effects [68, 267].

#### Vitamin E: modulatory effects on the aging immune system

Concerning immunity and in line with the already mentioned antioxidant function, tocols protect self-cells against damage during respiratory burst [268]. αT was able to reduce IL-8, IL-17 and CCL5 secretion in PHA-stimulated PBMCs, mediated by influencing prostaglandin E receptors 2 and 4 (EP2 and EP4) and thereby inducing the secretion of second messenger cyclic adenosine monophosphate (cAMP) [269]. Notably, reduced levels of peroxynitrite leading to the inhibited production and activity (not transcription) of prostaglandin E2 by macrophages and in turn to the decrease of the aging-associated enzyme cyclooxygenase (COX)−2 involved in inflammatory processes might play a role in these processes as well [270, 271].

Moreover, αT has been shown to inhibit the activity of protein kinase C (PKC) via activation of protein phosphatase 2 A (PP2A) resulting in the decreased production of superoxide in neutrophils and macrophages, the subdued proliferation of monocytes or macrophages among others, as well as the inhibition of extracellular signal-regulated kinase (ERK) 1/2- nuclear factor kappa-light-chain-enhancer of activated B cells (NF-κB) signaling cascade upon LPS treatment leading to a decreased COX-2 synthesis [236, 272–275]. In line with that, γ-tocotrienol was reported to inhibit the pro-inflammatory NF-κB pathway along with the release of cytokines IL-2, IFN-γ, IL-4 and IL-6 [276].

An effect of consuming *RRR*-αT or *all*-*rac-*αT on gene transcription, regarding certain differences between the natural and synthetic form, has been demonstrated in vivo, whereas the T cell-dependent expression of IL-2 and IL-10 among others was induced in mice being fed a high-tocopherol diet [277]. The endogenous αT metabolite 13-((2R)−6-hydroxy-2,5,7,8-tetramethylchroman-2-yl)−2,6,10-trimethyltridecanoic acid (α-T-13’-COOH) has been shown to inhibit 5lipoxygenase (5-LOX) in immune cells (involved in the biosynthesis of chemoattractant and vasoactive leukotrienes) and thereby decrease inflammation and bronchial hyper-reactivity in mice [278]. Along with initiating anti-inflammatory reactions, vitamin E might exert certain effects as part of an immune reaction such as T cell proliferation and differentiation upon supplementation above recommended levels (stimulation of proliferation and IL-2 secretion of activated naïve T cells most pronounced in the elderly), phagocytosis or antibody production [108, 267, 271].

Regarding T cell activity, vitamin E might be able to influence the aging-associated compromised recruitment of signaling proteins correlating with the development of an immune synapse between T cells and antibody presenting cells [279, 280]. In contrast, there have been reports about high-dose γT suppressing lymphocyte proliferation along with pro-inflammatory actions [267]. It must be noted that according to interindividual differences, the outcomes of vitamin E supplementation might differ in the elderly. Nonetheless, an increased dosage (100 mg vs. 50 mg vitamin E; six months intervention; healthy elderly 65–80 y/o) has been shown to influence cellular immune responsiveness including, for example, enhanced delayed-type hypersensitivity (DTH) and IL-2 production [281]. Meydani and colleagues reported similar findings concerning cellmediated immunity in healthy older adults upon supplementation of 800 mg dl-α-tocopheryl acetate for one month. Elevated DTH responses, increased IL-2 production and reduced prostaglandin E2 synthesis by PBMCs as well as plasma lipid peroxides were measured [282]. In contrast, Waart and colleagues could not find any effects of 100 mg dl-α-tocopheryl acetate ingested daily (three months; 65 years and older) regarding cellular (ex vivo stimulation with concanavalin A and PHA) and humoral (IgG and IgA) immune responses [283]. An in vivo study conducted in 2022 investigated how adding αT to the H1N1 influenza vaccine affects the diminished immune response in the elderly with regard to vaccine efficacy. Younger (6–8 weeks old) and older (16–20 weeks old) mice were immunized subcutaneously, resulting in improved IFN-γ and IL-4 responses, humoral immunity (HAI-titers), viral protection (decreased lung viral load), as well as survival rates in both age groups, whereas the effects regarding cytokine production was more pronounced in old mice, that vitamin E supplementation in combination with vaccination might increase the potency of the vaccine in the elderly [284].

Contrary to that, but in close relation to the described effects already mentioned for vitamin A, an observational prospective cohort study found no significant connection between differing micronutrient levels (vitamin E among others; no detectable insufficiency in any participant) and the serologic response to influenza vaccination measured by HAI titer, in 205 community-dwelling adults aged 65 and older, which contradicts the assumption that decreased levels of said micronutrients would be causing decreased HAI responses to vaccination [114]. Again, these observations are supported by the findings of Gardner et al. [115] which investigated the immune responses and plasma micronutrient levels (αT among others) in 61 healthy elderly (mean 81 y/o) compared to 27 young (mean 27 y/o) participants before and after influenza vaccination. The elderly showed comparingly low influenza vaccine induced proliferation and IFN-γ levels, as well as lower post-vaccination antibody titers, but these differences seemed to be independent from differing micronutrient levels [115]. Regarding viral infections, αT appears to be a potent mediator of (pulmonary) polymorphonuclear leukocyte (PMNs) responses (for example, decreased migration across lung epithelium or increased neutrophil elastase levels which elevates antimicrobial activity), making it applicable for the treatment of *Streptococcus pneumoniae* infections, especially in the elderly [285]. Hemilä [286] also examined how vitamin E affects the risk of pneumonia in male smokers aged 50–69 years based on the data of an intervention study (50 mg/d vitamin E; 5–8 years). Results showed a significant reduction in pneumonia incidence due to supplementation [286].

Research regarding the prevention of respiratory tract infections in elderly (nursing home residents) due to vitamin E supplementation appears to be heterogenous at times, especially regarding randomized controlled trials based on observatory outcomes such as number of incidence occurrence or number of antibiotic prescriptions [287].

In accordance with that, but using different methodology, observations contrary to previously described successful study outcomes have been made by van Amsterdam et al. [288] in the context of a randomized placebo-controlled study, in which the supplementation of 200 mg vitamin E daily for 15 months neither showed a significant impact on serum dehydroepiandrosterone (DHEA) sulfate ester nor neopterin, representing biomarkers of immunocompetence, in healthy elderly, which might explain the supposed failure of vitamin E protecting against acute respiratory infections as well as their correlating severity [288]. Despite the lack of statistical significance regarding the efficacy of vitamin E, which could be observed in some of the research articles, a positive trend towards protective effects concerning the incidence of common cold or overall infection rate has been described (compare [287]). However, it has to be noted that the immunologic impact of vitamin E supplementation regarding cytokine production might rely on genetic variables such as single nucleotide polymorphisms (SNPs), hence baseline production of cytokines prior to supplementation, as demonstrated in a double-blind, placebo-controlled intervention study on elderly subjects (mean 83 y/o) indicating, that individuals with specific genotypes (A/A and A/G) at TNF-α −308G > A (TNF-α SNP) may exert less TNF-α production, which suggests that anti-inflammatory action of vitamin E might be distinct for those being predisposed to higher inflammation rates, as the A allele correlates with higher TNF-α levels [289, 290]. These findings are supported by another study investigating the efficacy of vitamin E supplementation on the prevention of lower respiratory tract infections in elderly nursing home residents. The authors concluded that sex and specific SNPs at certain cytokine genes (for example, IL-10 −819G > A) play an important role regarding that matter [291]. Based on a nested case-control study within a multiethnic cohort and in relation to vitamin E and the risk of developing diseases, serum tocopherol levels representing adequate dietary vitamin E intake rather than high-dose supplementary levels might exert protective effects against developing non-Hodgkin lymphoma (NHL) [292]. A summary of these studies is provided in Table S1.

According to dietary intake surveys, the recommendations concerning αT intake are seldom met (population undersupply up to 75%), especially with regard of the elderly [293–296]. Contrary to that, a measurable deficiency rarely occurs under normal physiological conditions and a well-balanced diet, since adipose tissue serves as the main storage and vitamin E can be utilized up to several years [297, 298]. In contrast, severe vitamin E deficiency might be caused by disease such as lipid absorption abnormalities or polymorphisms in the liver-associated TTP resulting in reduced plasma levels, manifesting in, for example, acute peripheral neuropathy (degeneration of large calibre axons in sensory neurons), pigmented retinopathy, skeleton myopathy, spinocerebellar ataxia and immune system impairment, the progression of which can be decelerated and potentially reversed by tocopherol supplementation [133, 251, 298, 299]. Notwithstanding, as shown in numerous research articles, supplementation of vitamin E certainly has its benefits and might play a role in healthy aging. Short-term, high-dose tocopherol intake up to 300 mg/d does not cause major side effects, whereas persistent high-dose supplementation has been described to compromise blood clotting and might increase the chance of hemorrhagic strokes [300–302]. Commercially available supplements primarily contain esterified vitamin E due to increased oxidative stability (α-tocopheryl acetate and succinate; ≥ 67 mg (100 IU) *RRR*-αT) [251, 303]. Figure S2 provides a summary of the actual intake of vitamin D supplements compared with the reference values of D-A-CH, DGE, and NIH for men and women, based on the results of the German National Nutrition Survey II.

### Vitamin K

#### Vitamin K: general characteristics and physiological function

Vitamin K is the term for a group of compounds that is characterized by chlorophyll quinone bioactivity named after its main property - the promotion of blood coagulation [304]. Two forms, vitamin K_1_ (phylloquinone) and vitamin K_2_ (menaquinone) occur in nature in a wide variety of plant and animal products whereas vitamin K_3_ (menadione) is a synthetic analog and also intermediary product in the conversion of oral vitamin K_1_ into vitamin K_2_ [304, 305] (Fig. 1). Vitamins K_3_ and K_4_ (menadiol) have been considered to be synthetic water-soluble forms of vitamin K [306]. Vitamin K_1_ is mainly found in green, leafy or flowering vegetables and also in vegetable oils. Vitamin K_2_ describes a group of menaquinones (MK-n; n: number of isoprenyl residues) that is mainly found in meat, innards, eggs, dairy products, fermented foods and cheese. Natto from soybeans contains particularly high amounts of this vitamin [304, 307]. To date, MK-4 and MK-7 are the most studied menaquinones in the human diet. Vitamin K_2_ can also be provided by gut bacteria, even though in insufficient amounts [304, 308].

A growing number of studies show that vitamin K has many more functions than just its influence on blood clotting, some of which have long been neglected [304]. Vitamin K exerts beneficial effects regarding the antioxidant capacity, GI microbiome, epithelial development and function and helps protecting bones [304]. With regard to its bioactivity and function, the contribution to normal blood clotting and to the maintenance of normal bones were authorized as health claims in the European Union [95].

**Fig. 8:**
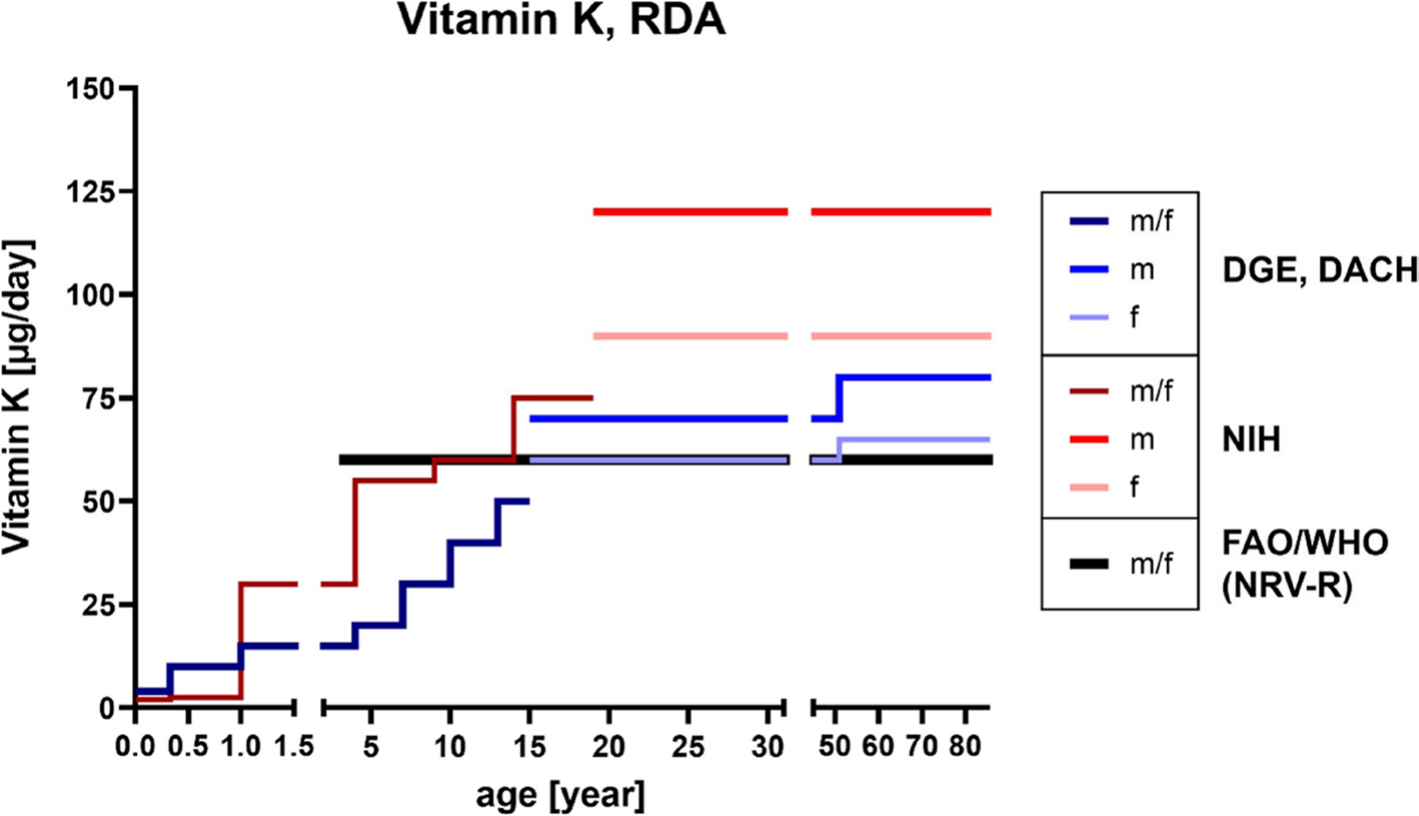
RDA reference values of vitamin K. References according to D-A-CH [87], DGE [313], NIH [83], and the NRVs-R of FAO/WHO [86]. D-A-CH, Deutschland, Austria, Confoederatio Helvetica (eng. GSA, Germany, Switzerland, Austria); DGE, Deutsche Gesellschaft für Ernährung (eng. German Nutrition Society); FAO, Food and Agriculture Organization; NIH, National Institutes of Health; NRV-R, Nutrient Reference Value-Requirement; RDA, Recommended Daily Allowance; WHO, World Health Organization

Protein S, a plasma glycoprotein encoded by the PROS1 gene, is also activated by γ-carboxylation and is involved in the inactivation of coagulation factors Va and VIIIa [309]. It seems to be important in local thrombosis prevention [310] and besides controlling coagulation, protein S promotes phagocytosis of apoptotic cells, cell survival, angiogenesis as well as vascular integrity and it also induces innate immunity [309]. In mice, these functions were largely lost following invalidation of the PROS1 gene [311]. Vitamin K_2_ was superior to vitamin K_1_ regarding inhibition of the cancer cell proliferation and induced cancer cell apoptosis and cell cycle arrest. When compared to synthetic and also more toxic vitamin K_3_, these effects were less pronounced [304, 312]. NRVs for vitamin K consumption amount to up to 60 µg [86], whereas the RDA is in the range of 125 µg/day being twice the amount (compare age groups 50 and older; D-A-CH, DGE and NIH reference values; Fig. 8). In contrast to the other fatsoluble vitamins, the German National Nutrition Survey II (2005–2007) did not provide any data concerning actual intake, intake below recommended values or supplementation.

#### Vitamin K: modulatory effects on the aging immune system

Recent studies have shown that the K vitamins also have positive effects on the immune system, including potential preventive and therapeutic effects on infectious diseases like asthma and COVID-19) but also inflammations in the context of type 2 diabetes mellitus, Alzheimer’s disease, Parkinson’s disease, cancer, aging and arteriosclerosis. These effects were also described for certain autoimmune diseases such as inflammatory bowel disease, type 1 diabetes mellitus, multiple sclerosis and rheumatoid arthritis [304]. It is becoming increasingly evident that there is a variety of vitamin K-dependent proteins (VKDPs) that exhibit immunomodulatory and anti-inflammatory effects increasing the complexity of vitamin K-related immune functions [304]. Vitamin K_2_, for example, prevented phosphorylation of NFκB inhibitor (IκB) by inhibiting IκB kinase (IKK) thereby decreasing cell cycle protein D1, thus suppressing the proliferation of cancer cells. Vitamin K_2_ also inhibited PKC kinase and PKD1 activity and therefore NF-κB activation [304, 314].

Asthma is caused by airway inflammation in connection with cytokines such as IL-4 and IL-13 [304, 315]. Vitamin K_2_ supplementation showed efficiency in asthma cases with varying degrees of severity [304, 316]. During infection, calcification of the lung is inhibited by matrix γ-carboxyglutamic acid protein (MGP) that is activated by vitamin K. High vitamin K levels correlate with high MGP levels and low IL-6 levels. Accordingly, vitamin K deficiency is connected to increased blood dephosphorylated-uncarboxylated MGP (dp-ucMGP) associated with reduced breathing capacity, increased risk of asthma andreduced protection of the elastic lung and vascular fibers which is a hallmark of COVID-19 [304, 317]. High dp-ucMGP and increased IL-6 levels together with low vitamin K levels have been identified as key factors in the inflammatory process and tissue destruction caused by COVID-19 [304, 317].

Due to the vitamin’s ability to activate both hepatic coagulation factors and the extrahepatic endothelial anticoagulant protein S, it seems plausible that vitamin K deficiency may be implicated in COVID-19 linking pulmonary and thromboembolic disease. This hypothesis is supported by a tight connection between extrahepatic vitamin K deficiency and poor outcome [317]. Therefore, Dofferhoff and colleagues hypothesized that the extrahepatic vitamin K depletion caused by pneumonia accelerates elastic fiber damage and thrombosis in severe COVID-19 due to impaired activation of MGP and endothelial protein S, respectively [310, 317]. Importantly, MK-7 levels were particularly low compared to non-COVID-19 pneumonia and healthy controls, indicating high consumption in extrahepatic tissues, especially the lungs [318].

Vitamin K_1_ and vitamin K_2_ treatments were investigated with T cells of mitogen-activated peripheral lymphocytes from healthy volunteers, but also from dialysis patients [319]. Interestingly, vitamin K_2_ dose-dependently suppressed the proliferation of PBMCs from both treatment groups stimulated by concanavalin A, while vitamin K_1_ showed no significant effects on PBMC proliferation. Both vitamins had no effect on the expression of most Th1/Th2/Th17 cytokines in activated PBMCs, with the exception of increased IL-4 expression in PBMCs from healthy volunteers and low T_reg_ levels in PBMCs from dialysis patients by vitamin K_2_ [319].

The anti-inflammatory mechanisms of vitamin K are not fully elucidated yet [306]. Activation of the multiprotein complex NLR family pyrin domain containing 3 (NLRP3) results in IL-1β and IL-18 secretion contributing to the pathogenesis of various human inflammatory diseases [306]. Interestingly, synthetic vitamins K_3_ and K_4_ are selective, potent inhibitors of NLRP3 inflammasome assembly by inhibiting the interaction between NLRP3 and the adaptor molecule apoptosis-associated speck-like protein containing a caspase recruitment domain (CARD; ASC) [306]. Accordingly, treatment with vitamin K_3_ or K_4_ attenuated the severity of inflammation in a murine peritonitis model [306]. Interestingly, vitamin K_1_ and K_2_ were not able to inhibit inflammasome activation supposedly due to their longer aliphatic chains [306].

Postmenopausal osteoporosis is characterized by more circulating activated T cells compared to healthy pre- and postmenopausal women and vitamin K_2_ could reduce the incidence of hip, vertebral and other fractures in these patients [320]. In this context, vitamin K_2_ (60 and 100 µM), but not vitamin K_1_, inhibited T cell proliferation [320]. Vitamin K_2_ had also suppressive effect on PBMCs of pediatric patients with atopic dermatitis at a dose of 10–100 µM by inhibiting the mitogen-activated protein kinase MEK1-ERK1/2 and SAPK/JNK signaling pathways [321]. It significantly attenuated the T cell mitogen-activated PBMC proliferation of atopic dermatitis patients and decreased the production of TNF-*α*, IL-10 and IL-17 A whereas IL-2 levels were increased [321].

Although dietary vitamin K deficiency is rare in healthy adults, it is common in infants and the elderly. For example, it has been shown that vitamin K production in the gut of patients taking broad-spectrum antibiotics is reduced by almost 74% probably due to the decline of gut bacteria [306]. Vitamin K is a very important prophylactic for all newborns, for example, to prevent bleeding as a side effect after vaccination, which mostly is vitamin K deficiency bleeding a serious condition in the neonatal period and early infancy [322]. In adults, vitamin K supplementation could also prevent severe COVID-19 infection in people at risk as a cheap and promising approach [310, 323]. The majority of patients receiving vitamin K antagonists is aged and the COVID-19 vaccine comirnaty decreased anticoagulation control in these patients [318].

Linneberg and colleagues investigated the hypothesis that low vitamin K status predicts mortality in COVID-19 patients in a cohort of 138 COVID-19 patients and 138 control subjects by measuring plasma dp-ucMGP to assess the loss of functional vitamin K in peripheral tissues [324]. Although low vitamin K status was associated with mortality in patients with COVID-19 in sex- and age-adjusted analyses, this correlation could not be confirmed after adjustment for co-morbidities [324]. Patients with inflammatory bowel disease suffer from damaged intestines resulting in malabsorption of vitamin K and thus vitamin K deficiency. As already mentioned, the latter is associated with several chronic inflammatory diseases [306]. Therefore, these patients should be screened for vitamin K deficiency besides vitamin D deficiency, as both conditions may be linked to the development of inflammatory bowel disease and in particular Crohn’s disease and the associated loss of bone health [325]. This is especially important since these patients are often affected by steroid use, reduced sunlight-exposure and inflammatory cytokines [325]. A summary of the aforementioned study outcomes is provided in Table S1.

## Conclusions

There is increasing evidence that all fat-soluble vitamins are involved in the regulation of the immune system. However, their molecular target structures and their contributions to biochemical pathways are very different and there is also a large discrepancy regarding the supply of vitamins in the population - particularly with regard to vulnerable groups such as the elderly.

Vitamin A, for example, exerts various effects on both the innate and adaptive immune system. It promotes the activity of lymphocytes and is involved in the maturation of DCs, Th cells and cytotoxic T cells. RA inhibits the development of Th1 cells and promotes the development of Th2 cells. 13-*cis*-RA increases the total count of lymphoid cells with Th cell surface markers and β-carotene affects the percentage of cells expressing NK cell markers. The supply situation in the German population is relatively good and only around 20% of the study participants are below the D-A-CH reference values.

In the elderly, physiological senescence could reduce RXR-β. While vitamin A has seemingly only moderate effects on the immune system in healthy study participants, it reduces disease progression and vitamin A deficiency results in increased susceptibility to bacterial and viral infections as well as inflammatory processes.

Vitamin D plays a very prominent role in immune responses and low vitamin D status is often described in the context of autoimmune and inflammatory diseases. Calcitriol appears to exert protective effects regarding inflammatory processes, promotes tolerogenic reactions and prevents excessive stimulation of the immune system. The immunogenic potential of vitamin D depends strongly on the expression of the VDR in many different immune cells. The effects are complex, as calcitriol, in addition to its immunostimulatory properties, for example, also suppresses the proliferation of certain immune cells and the production of IFN-γ.

Vitamin D deficiency is generally widespread and well over 90% of the German population are below the D-A-CH reference values and even 93% of the older Mexican population showed insufficient vitamin D levels despite being exposed to higher levels of UV-radiation. Insufficient vitamin D levels are not only associated with more frequent and more pronounced allergic rhinitis, low plasma calcifediol levels are also suspected to have a negative impact on the severity and mortality of COVID-19.

Although general dietary supplementation appears to have little effect in people with adequate serum vitamin D concentrations, its importance increases in infectious diseases and an inverse relationship between elevated calcifediol and CRP could be demonstrated [195]. Current studies emphasize the need for adequate consideration of serum calcifediol levels, especially in vulnerable groups.

Interestingly, vitamin E also shows also anti-inflammatory effects, influences the proliferation and differentiation of T cells as well as phagocytosis and antibody production. These effects were observed particularly when supplementation exceeded reference values, whereby on the other hand, very high doses can also suppress lymphocyte proliferation and even exhibit pro-inflammatory effects underpinning the complex influence on the human immune system. Despite the largely different doses among studies, further possible explanations for contradictory results could be gender-specific differences but also certain SNPs in cytokine genes. In relation to pneumonia and lung diseases in older people, there is some promising study data for supplementation and despite the heterogeneous data situation, a trend is also crystallizing regarding protective effects on the incidence of colds or general infection rates. Further, a role in healthy aging is discussed and short-term, high-dose tocopherol vitamin E of up to 300 mg/day did not cause major side effects.

While the D-A-CH reference values are largely reached in Germany, other studies show that an undersupply, particularly in vulnerable groups, is very common.

Recent studies have shown that vitamin K, whose role in the context of the immune system has so far been little researched, appears to have positive effects on the immune system. Particularly, preventive effects on various inflammatory and infectious diseases are discussed, as numerous VKPDs presumably have immune system-associated functions, which is, for example, illustrated by high dp-ucMGP plasma levels, which are related to reduced respiratory capacity with an unfavorable effect on asthma but also COVID-19. However, the mechanisms of vitamin K’s anti-inflammatory properties are not yet fully understood. Although nutritional vitamin K deficiency is rare in healthy adults, it is very common in older people and infants and therefore deserves special attention.

In summary, current data shows that the fat-soluble vitamins A, D, E and K all have an influence on the intact function of the immune system and in some cases also protect against its excessive activation. While vitamin D has long been known for its immune-promoting properties, there is already a very extensive body of data for vitamins A and E, while vitamin K has only recently become the focus of immunological interest in the context of severe respiratory diseases such as COVID-19, and it is clear that extensive research is still needed particularly for the latter vitamin in order to evaluate other previously unnoticed immune functions. It should also be noted that, according to the recommendations of the NIH, DGE and D-A-CH, only a very small proportion of the population is sufficiently supplied with vitamin D and that this deficiency appears to exist even in countries with high levels of sunlight, at least in vulnerable groups. While the supply of vitamins A and E in the general population is relatively good, their status in old age and in the event of illness should also be considered. For vitamin K, and in particular the biologically highly active vitamin K_2_, there is also a considerable lack of relevant recommendations, even with regard to the classic indications such as blood clotting and maintaining bone density, but especially in supporting the immune system.

Based on the available data, it is becoming increasingly clear that the fat-soluble vitamins are all involved in the maintenance of an adequate immune status and that the current vitamin levels, which can be partly determined by blood sampling, are an important parameter for the prevention and treatment of infectious diseases.

## Supplementary Information

Supplementary Material 1.

## Data Availability

No datasets were generated or analysed during the current study.

## Abbreviations

α/γT: α/γ-tocopherol
α‑TE: α‑tocopherol equivalents
α-TTP: α-tocopherol transfer protein
ABCA1: ATP binding cassette subfamily A member 1
ADCC: antibody-dependent cell-mediated cytotoxicity
APC: Antigen-presenting cell
ASC: Apoptosis-associated speck-like protein containing a CARD
ATRA: All‑*trans*‑retinoic acid
BCAA: Branched‑chain amino acid
cAMP: cyclic adenosine monophosphate
CARD: Caspase recruitment domain
CCL: CC-chemokine ligand
COX: Cyclooxygenase
CRP: C-reactive protein
COVID-19: Coronavirus disease 2019
CVID: Common variable immunodeficiency
D‑A‑CH: Deutschland, Austria, Confoederatio Helvetica
DC: Dendritic cell
DGE: Deutsche Gesellschaft für Ernährung
DHEA: Dehydroepiandrosterone
dp-ucMGP: dephosphorylated-uncarboxylated MGP
DTH: Delayed-type hypersensitivity
EFSA: European Food Safety Authority
eNOS: endothelial nitric oxide synthase
EP: Prostaglandin E receptor
ERK: Extracellular signal-regulated kinase
FAO: Food and Agriculture Organization
FGF23: Fibroblast growth factor 23
FoxP3: Forkhead box protein 3
GI: Gastrointestinal
GSA: Germany, Switzerland, Austria
GM-CSF: Granulocyte-macrophage colony-stimulating factor
HAI: Hemagglutination inhibition
HDL: High‑density lipoproteins
ICU: Intensive care unit
IFN-γ: Interferon-γ
Ig: immunoglobulin
IκB: NF-κB inhibitor
IKK: IκB kinase
IL: Interleukin
IP-10: IFN-inducible protein-10
IU: International unit
LDH: Lactate dehydrogenase
5-LOX: 5-lipoxygenase
LDL: Low-density lipoproteins
LPS: Lipopolysaccharide
MCP-1: Monocyte chemoattractant protein-1
MGP: Matrix γ-carboxyglutamic acid protein
MHC: Major histocompatibility complex
MIP-1β: Macrophage inflammatory protein-1β
MK-4: Menaquinone-4
mTOR: mammalian target of rapamycin
NF-κB: nuclear factor kappa-light-chain-enhancer of activated B cells
NHL: Non-Hodgkin lymphoma
NIH: National Institutes of Health
NK cell: Natural killer cell
NLR: NOD-like receptor
NLRP3: NLR family pyrin domain containing 3
NOD: Nucleotide-binding oligomerization domain
NRV: Nutrient Reference Values
NRV-R: Nutrient Reference Value-Requirement
PACAM: Programa de Alimentación Complementaria del Adulto Mayor
PBMC: Peripheral blood mononuclear cell
PHA: Phytohemagglutinin
PKC: Protein kinase C
PMN: Polymorphonuclear (leukocyte)
PP2A: Protein phosphatase 2A
PUFA: Polyunsaturated fatty acid
RA: Retinoic acid
RAE: Retinol activity equivalent
RAR: Retinoic acid receptor
RBP: Retinol-binding protein
RBPR: Retinol-binding protein receptor
RDA: Recommended Dietary Allowance
RE: Retinol equivalent
ROS: Reactive oxygen species
RXR: Retinoid X receptor
SARS-CoV-2: Severe acute respiratory syndrome coronavirus type 2
SASP: Senescence-associated secretory phenotype
SNP: Single nucleotide polymorphism
TGF: Transforming growth factor
Th cell: T helper cell
TLR: Toll-like receptor
TNF-α: Tumor necrosis factor α
T_reg_: Regulatory T cell
UVB: Ultraviolet B
VDR: Vitamin D receptor
VDRE: Vitamin D response element
VEGF: Vascular endothelial growth factor
VKDP: Vitamin K-dependent protein
WHO: World Health Organization
