# Supplementary material for: Exploring the link between fat-soluble vitamins and aging-associated immune system status: a literature review
Source: Immun Ageing. 2025 Feb 17;22:8. doi: 10.1186/s12979-025-00501-3 (PMC11831837; doi:10.1186/s12979-025-00501-3)
Supplement: Supplementary file 1 — Supplementary Material 1. [file 12979_2025_501_MOESM1_ESM.pdf]

Supplementary figures and table

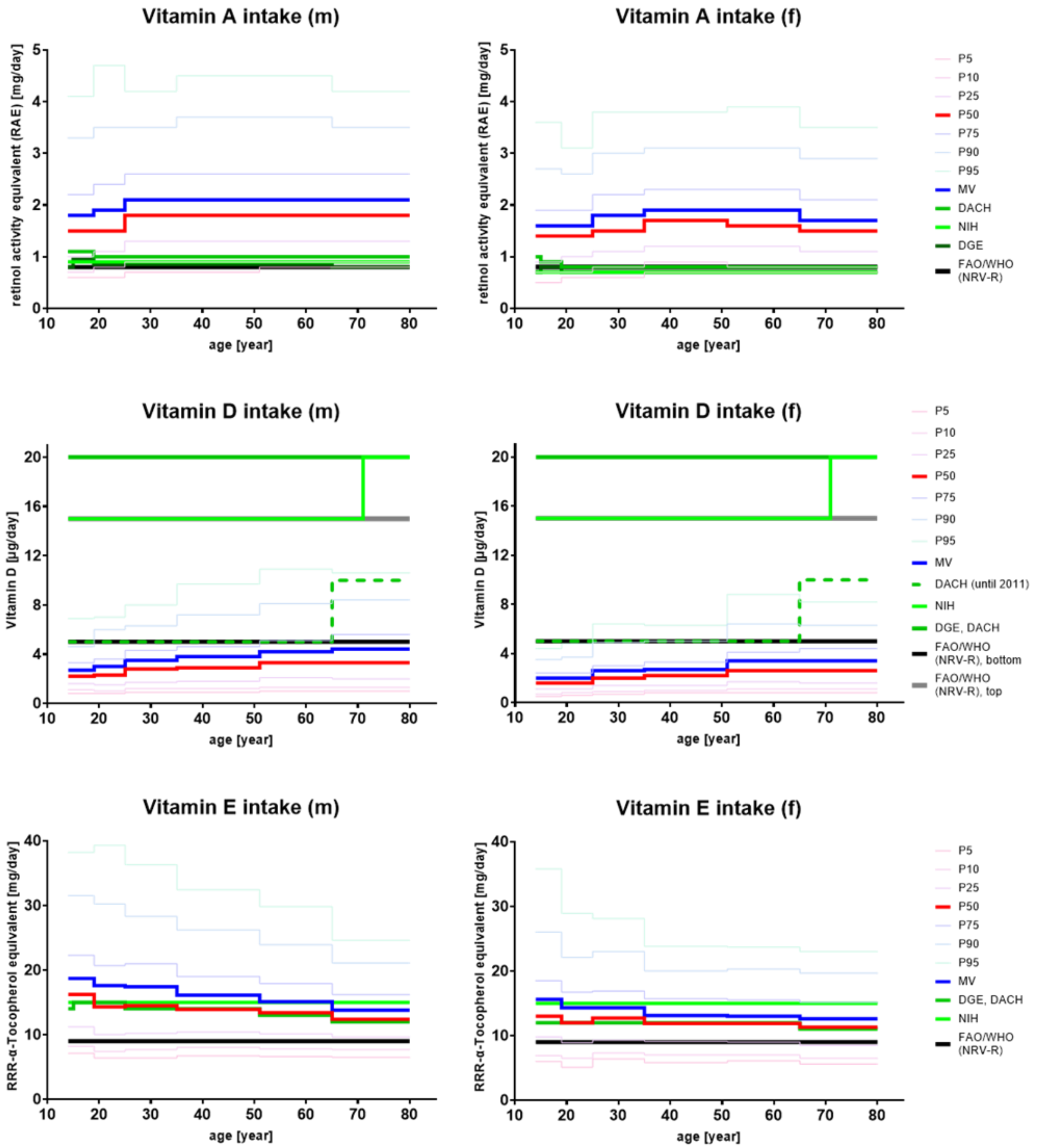

Figure S1: Results of the German National Nutrition Survey II (1) of actual vitamin intake compared with the reference values of D-A-CH (2), DGE (3–5), NIH (6–8), and the NRVs-R of FAO/WHO (9) for men (m, n = 7093) and women (f, n = 8278) of different ages. D-A-CH, Deutschland, Austria, Confoederatio Helvetica (eng. GSA, Germany, Switzerland, Austria); DGE, Deutsche Gesellschaft für Ernährung (German Nutrition Society); FAO, Food and Agriculture Organization; NIH, National Institutes of Health; NRV-R, Nutrient Reference Value-Requirement; RDA, Recommended Daily Allowance; WHO, World Health Organization.

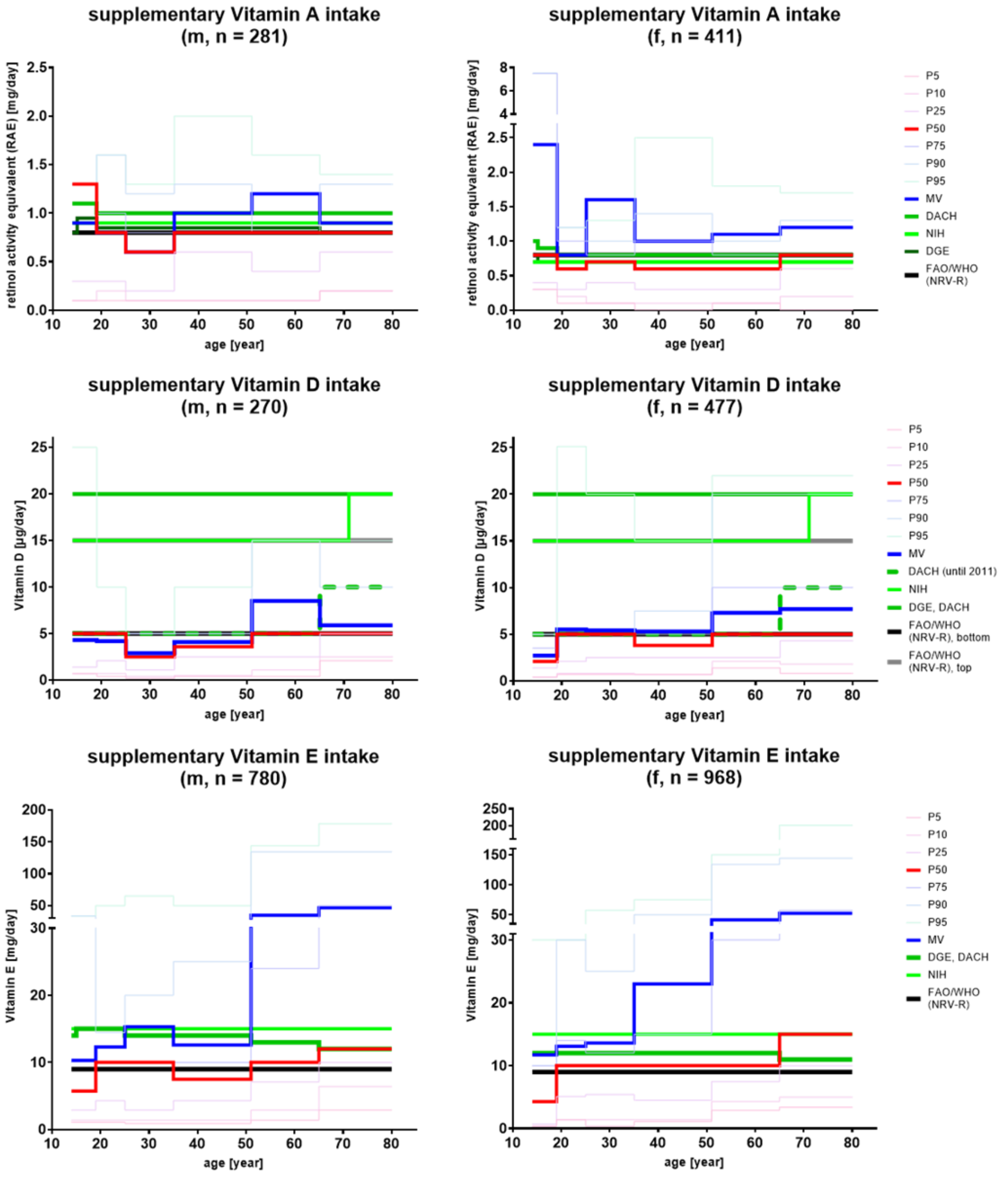

Figure S2: Results of the german National Nutrition Survey II (10) of actual supplemantary vitamin intake compared with the reference values of D-A-CH (2), DGE (3–5), NIH (6–8), and the NRVs-R of FAO/WHO (9) for men and women of different ages. D-A-CH, Deutschland, Austria, Confoederatio Helvetica (eng. GSA, Germany, Switzerland, Austria); DGE, Deutsche Gesellschaft für Ernährung (German Nutrition Society); FAO, Food and Agriculture Organization; NIH, National Institutes of Health; NRV-R, Nutrient Reference Value-Requirement; RDA, Recommended Daily Allowance; WHO, World Health Organization.

Table S1: Relevant human studies on the status and supplementation of fat-soluble vitamins and their effects on health and disease in the elderly.

Compound	Observation	Disease	Age of Participants (years)	Number of Participants	Reference
Vitamin A	No Association Between Vitamin A Levels and Serologic Response to Influenza Vaccine	Healthy	≥ 65	205	(11)
	No Effect of Vitamin A Levels on Immune Response to Influenza Vaccine	Healthy	70 – 95	61	(12)
	No Effect of Supplementation on Incidence of Antibiotic-treated Bacterial Infections	Healthy	75.7 ± 12.2	53	(13)
	Inverse Correlation Between Disease Severity as well as Mortality and Plasma Vitamin A Levels	COVID-19	30 – 82	40	(14)
	Decreased Plasma Vitamin A Levels, Supplementation → TNF-α ↓ and IL-10 ↑	CVID	21 – 67	20	(15)
	Supplementation → T_{reg} Expression ↑, Effector T Cell Expression ↓	Atherosclerosis	38 – 69	31	(16)
	Supplementation → General Immune Stimulating Effects	Unresectable Squamous Cell Carcinoma of the Lung	65 – 77	9	(17)
	37% of Patients with Vitamin A Deficiency, 23% with Decreased Serum Levels in Patients with Severe Symptoms	COVID-19	≥ 18	155	(18)
	Supplementation → No Effect on Influenza Vaccine Response	Healthy	72 ± 5	19	(19)

Vitamin D	Inverse Association Between Elevated Serum Calcifediol and Markers of Systemic Inflammation	Healthy	Mean: 64.9	9,649	(20)
	Supplementation → No Effect on Hospitalizations due to Infection, Reduction in Extended Hospitalizations	Healthy	60 – 84	21,315	(21)
	Supplementation → No Effect on Cytokine Concentration	Healthy	≥ 64	202	(22)
	Decreased Serum Calcifediol → Increased Symptom Severity and Mortality as well as Worsened Disease Outcome	COVID-19	65 ± 13	42	(23)
	Decreased Serum Calcifediol → Increased Risk of Hospitalized Pneumonia	Healthy	53 – 73	1,421	(24)
	Supplementation → No Effect on Infection Parameters	COVID-19	55.3 ± 14.2	101	(25)
Vitamin E	Adequate Vitamin E Intake → Protective Effects Against Developing NHL	Healthy	70 ± 7.4	609	(26)
	Immunologic Effects of Supplementation Depending on SNPs at Certain Cytokine Genes	Healthy<br>Healthy<br>Healthy	68 – 100<br>65 – 100<br>65 – 102	110<br>110<br>500	(27–29)
	Supplementation → No Significant Effect on Biomarkers of Immune Competence	Healthy	> 60	100	(30)
	Supplementation → Reduction in Pneumonia Incidence in Male Smokers	Healthy	50 – 69	7,469	(31)
	No Influence of Plasma Levels on Influenza Vaccine Response	Healthy	70 – 95	61	(12)
	Supplementation → Enhanced DTH and IL-2 production	Healthy	65 – 80	161	(32)
	Supplementation → Increased DTH Responses, Increased IL-2 Production, Decreased Prostaglandin E2 Synthesis and Plasma Lipid Peroxides	Healthy	≥ 60	32	(33)

Vitamin K	No Correlation Between Low Vitamin K Status and Patient Mortality	COVID-19	68.4 ± 15.6	138	(34)
	COVID-19 Infection → Decreased MK7 and Increased MK4 levels	COVID-19	47 – 81	316	(35)

COVID-19: Coronavirus disease 2019; CVID: common variable immunodeficiency; DTH: delayed-type hypersensitivity; IL: interleukin; NHL: non-Hodgkin lymphoma; MK: menaquinone; SNP: single nucleotide polymorphism; TNF-α: Tumor necrosis factor α

Literature

1. Max Rubner-Institut. Nationale Verzehrsstudie II. Ergebnisbericht, Teil 2: Die bundesweite Befragung zur Ernährung von Jugendlichen und Erwachsenen [p. 249]. MRI; 2008 [cited 2024 Jul 24]. Available from: URL: https://www.openagrar.de/receive/bmelv_mods_00000135.

2. Deutsche Gesellschaft für Ernährung; Österreichische Gesellschaft für Ernährung; Schweizerische Gesellschaft für Ernährung. Referenzwerte für die Nährstoffzufuhr. 2. Auflage, 5. aktualisierte Ausgabe. Bonn: Deutsche Gesellschaft für Ernährung; 2019.

3. Vitamin A: DGE; 2024:43:35. Available from: URL: http://www.dge.de/wissenschaft/referenzwerte/vitamin-a/.

4. Vitamin D: DGE; 2024:00:35. Available from: URL: http://www.dge.de/wissenschaft/referenzwerte/vitamin-d/.

5. Vitamin E: DGE; 2024:08:27. Available from: URL: http://www.dge.de/wissenschaft/referenzwerte/vitamin-e/.

6. Institute of Medicine (U.S.). Dietary Reference Intakes for Vitamin A, Vitamin K, Arsenic, Boron, Chromium, Copper, Iodine, Iron, Manganese, Molybdenum, Nickel, Silicon, Vanadium, and Zinc: A Report of the Panel on Micronutrients. Washington, D.C., USA: National Academies Press; 2002.

7. Institute of Medicine (U.S.). Dietary Reference Intakes for Calcium and Vitamin D. Washington, D.C.: National Academies Press; 2011. Available from: URL: http://www.nap.edu/catalog/13050.

8. Institute of Medicine (U.S.). Dietary reference intakes for vitamin C, vitamin E, selenium, and carotenoids: A report of the Panel on Dietary Antioxidants and Related Compounds, Subcommittees on Upper Reference Levels of Nutrients and Interpretation and Uses of Dietary Reference Intakes, and the Standing Committee on the Scientific Evaluation of Dietary Reference Intakes, Food and Nutrition Board, Institute of Medicine. Washington, D.C., USA: National Academy Press; 2000.

9. Lewis J. Codex nutient reference values: Especially for vitamins, minerals and protein. Rome: FAO and WHO; 2019.

10. Max Rubner-Institut. Nationale Verzehrsstudie II. Ergebnisbericht, Teil 2: Die bundesweite Befragung zur Ernährung von Jugendlichen und Erwachsenen [p. 266]. MRI; 2008 [cited 2024 Jul 24]. Available from: URL: https://www.openagrar.de/receive/bmelv_mods_00000135.

11. Sundaram ME, Meydani SN, Vandermause M, Shay DK, Coleman LA. Vitamin E, vitamin A, and zinc status are not related to serologic response to influenza vaccine in older adults: an observational prospective cohort study. Nutr Res 2014; 34(2):149–54.

12. Gardner EM, Bernstein ED, Popoff KA, Abrutyn E, Gross P, Murasko DM. Immune response to influenza vaccine in healthy elderly: lack of association with plasma beta-carotene, retinol, alpha-tocopherol, or zinc. Mechanisms of Ageing and Development 2000; 117(1-3):29–45. Available from: URL: https://www.sciencedirect.com/science/article/pii/s0047637400001342.

13. Murphy S, West KP, Greenough WB, Cherot E, Katz J, Clement L. Impact of vitamin A supplementation on the incidence of infection in elderly nursing-home residents: a randomized controlled trial. Age Ageing 1992; 21(6):435–9.

14. Tepasse P-R, Vollenberg R, Fobker M, Kabar I, Schmidt H, Meier JA et al. Vitamin A Plasma Levels in COVID-19 Patients: A Prospective Multicenter Study and Hypothesis. Nutrients 2021; 13(7).

15. Aukrust P, Müller F, Ueland T, Svardal AM, Berge RK, Frøland SS. Decreased vitamin A levels in common variable immunodeficiency: vitamin A supplementation in vivo enhances immunoglobulin production and downregulates inflammatory responses. Eur J Clin Invest 2000; 30(3):252–9.

16. Mottaghi A, Salehi E, Keshvarz A, Sezavar H, Saboor-Yaraghi A-A. The influence of vitamin A supplementation on Foxp3 and TGF-β gene expression in atherosclerotic patients. J Nutrigenet Nutrigenomics 2012; 5(6):314–26.

17. Micksche M, Cerni C, Kokron O, Titscher R, Wrba H. Stimulation of immune response in lung cancer patients by vitamin A therapy. Oncology 1977; 34(5):234–8.

18. Al-Saleh I, Alrushud N, Alnuwaysir H, Elkhatib R, Shoukri M, Aldayel F et al. Essential metals, vitamins and antioxidant enzyme activities in COVID-19 patients and their potential associations with the disease severity. Biometals 2022; 35(1):125–45.

19. Goncalves-Mendes N, Talvas J, Dualé C, Guttmann A, Corbin V, Marceau G et al. Impact of Vitamin D Supplementation on Influenza Vaccine Response and Immune Functions in Deficient Elderly Persons: A Randomized Placebo-Controlled Trial. Front Immunol 2019; 10:65.

20. Liefaard MC, Ligthart S, Vitezova A, Hofman A, Uitterlinden AG, Kiefte-de Jong JC et al. Vitamin D and C-Reactive Protein: A Mendelian Randomization Study. PLoS One 2015; 10(7):e0131740.

21. Pham H, Waterhouse M, Baxter C, Romero BD, McLeod DS, Armstrong BK et al. Vitamin D supplementation and hospitalization for infection in older adults: A post-hoc analysis of data from the Australian D-Health Trial. The American Journal of Clinical Nutrition 2023; 117(2):350–6.

22. Barnes MS, Horigan G, Cashman KD, Hill TR, Forsythe LK, Lucey AJ et al. Maintenance of wintertime vitamin D status with cholecalciferol supplementation is not associated with alterations in serum cytokine concentrations among apparently healthy younger or older adults. J Nutr 2011; 141(3):476–81.

23. Carpagnano GE, Di Lecce V, Quaranta VN, Zito A, Buonamico E, Capozza E et al. Vitamin D deficiency as a predictor of poor prognosis in patients with acute respiratory failure due to COVID-19. J Endocrinol Invest 2021; 44(4):765–71.

24. Aregbesola A, Voutilainen S, Nurmi T, Virtanen JK, Ronkainen K, Tuomainen T-P. Serum 25-hydroxyvitamin D3 and the risk of pneumonia in an ageing general population. J Epidemiol Community Health 2013; 67(6):533–6.

25. Fernandes AL, Murai IH, Reis BZ, Sales LP, Santos MD, Pinto AJ et al. Effect of a single high dose of vitamin D3 on cytokines, chemokines, and growth factor in patients with moderate to severe COVID-19. Am J Clin Nutr 2022; 115(3):790–8.

26. Morimoto Y, Ollberding NJ, Cooney RV, Wilkens LR, Franke AA, Le Marchand L et al. Prediagnostic serum tocopherol levels and the risk of non-hodgkin lymphoma: the multiethnic cohort. Cancer Epidemiol Biomarkers Prev 2013; 22(11):2075–83.

27. Belisle SE, Leka LS, Dallal GE, Jacques PF, Delgado-Lista J, Ordovas JM et al. Cytokine response to vitamin E supplementation is dependent on pre-supplementation cytokine levels. Biofactors 2008; 33(3):191–200.

28. Belisle SE, Leka LS, Delgado-Lista J, Jacques PF, Ordovas JM, Meydani SN. Polymorphisms at cytokine genes may determine the effect of vitamin E on cytokine production in the elderly. J Nutr 2009; 139(10):1855–60.

29. Belisle SE, Hamer DH, Leka LS, Dallal GE, Delgado-Lista J, Fine BC et al. IL-2 and IL-10 gene polymorphisms are associated with respiratory tract infection and may modulate the effect of vitamin E on lower respiratory tract infections in elderly nursing home residents. Am J Clin Nutr 2010; 92(1):106–14.

30. van Amsterdam J, van der Horst-Graat J, Bischoff E, Steerenberg P, Opperhuizen A, Schouten E. The effect of vitamin E supplementation on serum DHEA and neopterin levels in elderly subjects. Int J Vitam Nutr Res 2005; 75(5):327–31.

31. Hemilä H. Vitamin E administration may decrease the incidence of pneumonia in elderly males. Clin Interv Aging 2016; 11:1379–85.

32. Pallast EG, Schouten EG, Waart FG de, Fonk HC, Doekes G, Blomberg BM von et al. Effect of 50- and 100-mg vitamin E supplements on cellular immune function in noninstitutionalized elderly persons. 0002-9165 1999; 69(6):1273–81.
33. Meydani SN, Barklund MP, Liu S, Meydani M, Miller RA, Cannon JG et al. Vitamin E supplementation enhances cell-mediated immunity in healthy elderly subjects. 0002-9165 1990; 52(3):557–63.
34. Linneberg A, Kampmann FB, Israelsen SB, Andersen LR, Jørgensen HL, Sandholt H et al. The Association of Low Vitamin K Status with Mortality in a Cohort of 138 Hospitalized Patients with COVID-19. Nutrients 2021; 13(6).
35. Mangge H, Prueller F, Dawczynski C, Curcic P, Sloup Z, Holter M et al. Dramatic Decrease of Vitamin K2 Subtype Menaquinone-7 in COVID-19 Patients. Antioxidants (Basel) 2022; 11(7).
